# Deuteration Induced Electron‐Phonon Coupling Modulation: Suppressing Energy Dissipation and Enhancing Carrier Separation in Organic Photocatalysis

**DOI:** 10.1002/advs.202516740

**Published:** 2025-11-19

**Authors:** Hongli Sun, Zirui Zhou, Yunfei Ma, Qingzhu Xu, Yanglong Liao, Cheng Han, Yue Zheng, Xiaosong Cao, Yu Wang, Zutao Fan, Jianfeng Zhao, Chenliang Su, Fengtao Fan, Bin Liu

**Affiliations:** ^1^ International Collaboration Laboratory of 2D Materials for Optoelectronics Science and Technology of Ministry of Education Institute of Microscale Optoelectronics Shenzhen University Shenzhen 518060 China; ^2^ State Key Laboratory of Radio Frequency Heterogeneous Integration Shenzhen University Shenzhen 518060 China; ^3^ College of Chemistry and Chemical Engineering Yangzhou University Yangzhou 225002 China; ^4^ Shenzhen Key Laboratory of New Information Display and Storage Materials College of Materials Science and Engineering Shenzhen University Shenzhen 518060 China; ^5^ State Key Laboratory of Catalysis Dalian National Laboratory for Clean Energy The Collaborative Innovation Centre of Chemistry for Energy Materials (iChEM) Dalian Institute of Chemical Physics Chinese Academy of Sciences Dalian 116023 China; ^6^ Department of Materials Science and Engineering City University of Hong Kong Hong Kong SAR 999077 China; ^7^ Department of Chemistry Hong Kong Institute for Clean Energy (HKICE) & Center of Super‐Diamond and Advanced Films (COSDAF) City University of Hong Kong Hong Kong SAR 999077 China

**Keywords:** 4CzIPN, deuteration, electron‐phonon coupling, non‐radiative energy dissipation, organic photocatalysts

## Abstract

The electron‐phonon coupling in organic photocatalysts offers a great opportunity for tuning carrier behaviors and energy dissipation toward improving photocatalytic efficiency. Adopting strategies to tailor electron‐phonon coupling and revealing the underlying mechanism are therefore essential for the development of high‐efficiency organic photocatalysts. In this work, an isotope substitution strategy is developed by replacing H in high‐frequency C─H oscillators with D to tune the electron‐phonon coupling strength of both 1,2,3,5‐tetrakis(carbazol‐9‐yl)‐4,6‐dicyanobenzene (4CzIPN) and its polymeric derivative. The fitted Huang–Rhys factors exhibit an ≈1.7‐fold increase upon isotopic deuteration, revealing enhanced electron‐phonon coupling. Comprehensive studies demonstrate that the deuteration strategy can effectively lower exciton binding energy, promote exciton dissociation, and suppress non‐radiative energy dissipation, therefore leading to an improved efficiency toward photocatalytic hydrogen evolution. This study highlights the crucial role of electron‐phonon coupling on photocatalytic systems and presents a novel regulatory strategy for designing high‐efficiency organic photocatalysts.

## Graphical abstract

During charge transport, photogenerated carriers exchange energy with lattice vibrations, influencing photocatalytic performance. The deuteration strategy enhances exciton dissociation and suppresses non‐radiative energy loss, significantly improving photocatalytic hydrogen evolution.

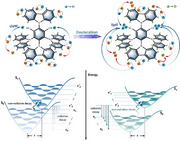

## 1 Introduction

In photocatalytic systems, the generation, migration, and separation of charge carriers in photocatalysts are critically important for photocatalytic performance, which can be readily tuned by the structural factors at the atomic and molecular scales. Traditionally, the structure tailor mainly focused on the electronic properties of the photocatalysts via vacancy engineering, facet engineering, doping, heterostructuring, etc.^[^ ^1^, ^2^, ^3^, ^4^, ^5^ ^]^ However, in photocatalysis, the transfer of photogenerated charge carriers is not solely governed by the electronic energy levels of the photocatalysts. It should be noted that the constituent atoms in photocatalysts are not completely stationary but instead vibrate around their equilibrium positions. The collective vibrations of atoms in photocatalysts can introduce quantized phonon energy levels, which shall influence the migration of charges during photocatalysis. In the charge transport process, the photogenerated charge carriers are likely to engage in energy transfer with vibrating atoms, thus affecting the photocatalytic performance. However, although crucial, the electron‐phonon interactions and their impacts on the thermodynamics and kinetics of charge carriers are overlooked for a long time in photocatalytic systems.^[^ ^6^ ^]^

In recent years, organic photocatalysts have made significant advancements in photocatalytic water splitting, hydrogen peroxide generation, CO_2_ conversion, organic synthesis, etc.^[^ ^7^, ^8^, ^9^, ^10^ ^]^ Constructing donor‐acceptor (D‐A) units offers a widely used strategy for improving the spatial separation of photogenerated electrons and holes in organic photocatalysts.^[^ ^11^, ^12^ ^]^ Unfortunately, the D‐A structure still possesses a high likelihood of non‐radiative energy dissipation, restricting the photocatalytic efficiency.^[^ ^13^, ^14^, ^15^, ^16^ ^]^ Toward alleviating the problem of non‐radiative energy dissipation, some strategies were developed, including changing the planar configuration to weaken interlayer energy transfer,^[^ ^17^, ^18^ ^]^ introducing a rigid structure to restrict molecule rotation,^[^ ^19^, ^20^ ^]^ etc. However, these approaches suffer from unavoidable changes of the original physical and chemical properties of organic photocatalysts, facing uncontrollable variables.

For organic photocatalysts bearing rich hydrogen atoms, the isotope substitution strategy by replacing hydrogen (H) with deuterium (D), which doubles the atomic mass, can lead to significant changes in bond vibration frequencies, and at the same time maintain the original physical and chemical properties of the material.^[^ ^21^, ^22^ ^]^ This offers a promising strategy for tailoring electron‐phonon interactions. When X─H bonds (X = O, N, C) in organic photocatalysts were replaced by X─D bonds, the X─D bonds would vibrate at lower frequencies due to the isotope effect, which could alter the overall vibrational system and affect the interactions between electrons and phonons, consequently changing the dynamic behaviors and lifetime of photogenerated charge carriers.^[^ ^23^, ^24^, ^25^, ^26^ ^]^

In view of the electron‐phonon interactions and energy dissipation in organic photocatalysts with D‐A structures, 1,2,3,5‐tetrakis(carbazol‐9‐yl)‐4,6‐dicyanobenzene (4CzIPN) and its polymeric derivative (poly‐4CzIPN) were chosen as the model organic photocatalysts to study the deuteration effects on electron‐phonon coupling and photocatalytic performance, given their great potential in photocatalytic hydrogen production, organic synthesis, etc.^[^ ^14^, ^27^, ^28^, ^29^, ^30^ ^]^ Through deuterium substitution, both D‐4CzIPN and poly‐D‐4CzIPN exhibit enhanced photocatalytic hydrogen evolution rate under visible light illumination. Comprehensive studies indicate that the deuterated photocatalysts possess weakened C─D bond stretching and bending energy, and increased electron‐phonon interactions. This tailor of electron‐phonon interactions in deuterated photocatalysts effectively promotes the dissociation of excitons and suppresses the non‐radiative decay process of photogenerated charge carriers, greatly promoting the photocatalytic hydrogen evolution performance.

## 2 Results and Discussion

**Figure 1:**
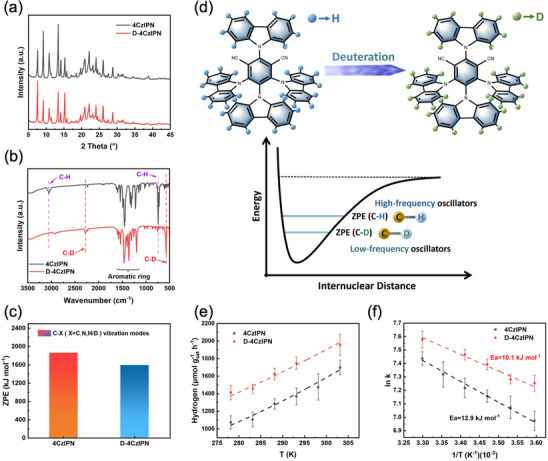
a) XRD patterns. b) FTIR spectra. c) The effects of deuteration on the ZPEs. d) A schematic diagram showing the deuteration strategy and its effects on ZPEs between the non‐deuterated and deuterated molecules. e) The hydrogen production rate of 4CzIPN and D‐4CzIPN recorded in the temperature range of 5–30 °C. f) Arrhenius diagram of 4CzIPN and D‐4CzIPN.

The 4CzIPN/D‐4CzIPN was synthesized via a one‐step nucleophilic substitution reaction between carbazole and fluorobenzonitrile (Scheme S1, Supporting Information).^[^ ^31^, ^32^ ^]^ It could be observed in the ^13^C‐NMR and ^1^H‐NMR spectra that the hydrogen intensity of specific chemical shifts decreased after deuterium substitution (Appendices SA1–SA4, Supporting Information), indicating that deuterium substitution successfully reduced the amount of hydrogen. The actual molecular weight of D‐4CzIPN(C_56_D_33_N_6_Na^+^) was determined to be 843 g mol^−1^ by mass spectrometry (Appendices SA5 and SA6, Supporting Information), further demonstrating successful deuteration (98% D). X‐ray diffraction patterns (**Figure** **1**a) of 4CzIPN and D‐4CzIPN exhibited the same crystalline features, suggesting that the deuteration strategy would not alter the crystalline structure of 4CzIPN. Furthermore, the similar C 1s and N 1s core level X‐ray photoelectron spectroscopy spectra (Figure S1, Supporting Information) demonstrated that deuteration had a negligible influence on the elemental valence states. As shown in the Fourier transform infrared spectroscopy spectra (FTIR, Figure 1b), 4CzIPN displayed the typical aromatic ring skeleton vibrations in the wavenumber range of 1700–1100 cm^−1^, the C≡N stretching vibration at 2234 cm^−1^, and the C─H stretching vibration at 3056 cm^−1^. In comparison, significant changes were observed in the C─D bond vibrations of D‐4CzIPN after deuteration. The stretching vibration at 3056 cm^−1^ and the bending vibration at 741 cm^−1^ of the C─H bond shifted to 2278 and 573 cm^−1^, respectively, in D‐4CzIPN, equaling to an energy reduction of 2.2 and 0.48 kcal mol^−1^, respectively, consistent with the vibration frequency ratio of the valence bond after the isotope substitution (*ν*_C–H_:*ν*_C–D_ ≈$\sqrt{2}$).^[^ ^24^, ^33^, ^34^ ^]^ Concomitantly, a frequency reduction in the fingerprint region (1700–1100 cm^−1^) was also observed, implying a more compact distribution of vibrational energy states in D‐4CzIPN. Therefore, deuteration could decrease the stretching and bending energy of the entire molecule, especially suppressing the high‐frequency C─H stretching and bending vibrations. Theoretical calculation further evidenced the isotope effect on zero‐point energy (ZPE) of 4CzIPN (Figure 1c). It was found that, by replacing hydrogen with deuterium, the ZPE value was notably reduced owing to the suppression of high‐frequency vibrations. Considering the effects of deuteration on vibrational energy, a schematic diagram of the deuteration strategy is presented in Figure 1d, where the deuterated catalyst possesses a relatively lower population of high vibrational energy levels owing to the suppression of high‐frequency vibration modes. As shown in Figure 1e, the deuterated D‐4CzIPN displayed an obviously improved photocatalytic hydrogen evolution activity across all temperatures (5–30 °C) examined as compared to 4CzIPN. By fitting the data according to the Arrhenius equation,^[^ ^35^, ^36^ ^]^

(1) $$k=Ae^{-\frac{Ea}{RT}}$$

where *k* is the reaction rate constant, *A* is the pre‐exponential factor, *E_a_* is the apparent activation energy, *R* is the gas constant, and *T* is the absolute temperature (K), the *E_a_* of 4CzIPN and D‐4CzIPN were determined to be 12.9 and 10.1 kJ mol^−1^, respectively (Figure 1f), matching well with the improved photocatalytic activity of D‐4CzIPN. To determine the structure stability of the deuterated C─D bonds in D‐4CzIPN during the photocatalytic hydrogen evolution reaction, photocatalytic hydrogen evolution cycling tests for D‐4CzIPN and 4CzIPN were performed (Figure S2, Supporting Information). Figure S3 (Supporting Information) compared the FTIR spectra of the photocatalysts before and after five cycles of photoreaction. The results indicated that the structure of D‐4CzIPN remained nearly unchanged after five cycles of photoreaction, demonstrating its excellent photocatalytic stability. To determine whether H_2_ originated from water or sacrificial agent, and to rule out the potential influence of the platinum source (chloroplatinic acid hexahydrate) on the experimental results, isotope labeling experiments with D‐4CzIPN in the absence of triethanolamine (TA) were first conducted (Figures S4 and S5, Supporting Information). As illustrated in Figure S5 (Supporting Information), when H_2_O was used as the solvent, a distinct signal at molecular mass 2 (H_2_) was observed. However, when H_2_O was replaced with deuterated water (D_2_O), the signal at mass 2 disappeared, and a new signal at molecular mass 4 (D_2_) was detected instead, confirming that water served as the hydrogen source. Subsequently, isotope labeling experiments with D‐4CzIPN in the presence of TA were performed, as summarized in Figures S6 and S7 (Supporting Information). When TA was added to the H_2_O solution, only the signal corresponding to molecular mass 2 (H_2_) was detected. In contrast, when D_2_O was used as the solvent, signals at molecular masses 2, 3, and 4 – corresponding to H_2_, HD, and D_2_, respectively – were all observed. Notably, the peak area of H_2_ decreased dramatically upon switching to D_2_O, and the signal intensities of both H_2_ and HD were substantially lower than that of D_2_. These results indicated that in the photocatalytic hydrogen evolution process, water acted as the main hydrogen source, while TA served only as a minor contributor. Additionally, throughout the experiments, no signal at molecular mass 32 was detected, confirming the absence of O_2_ generation.

**Figure 2:**
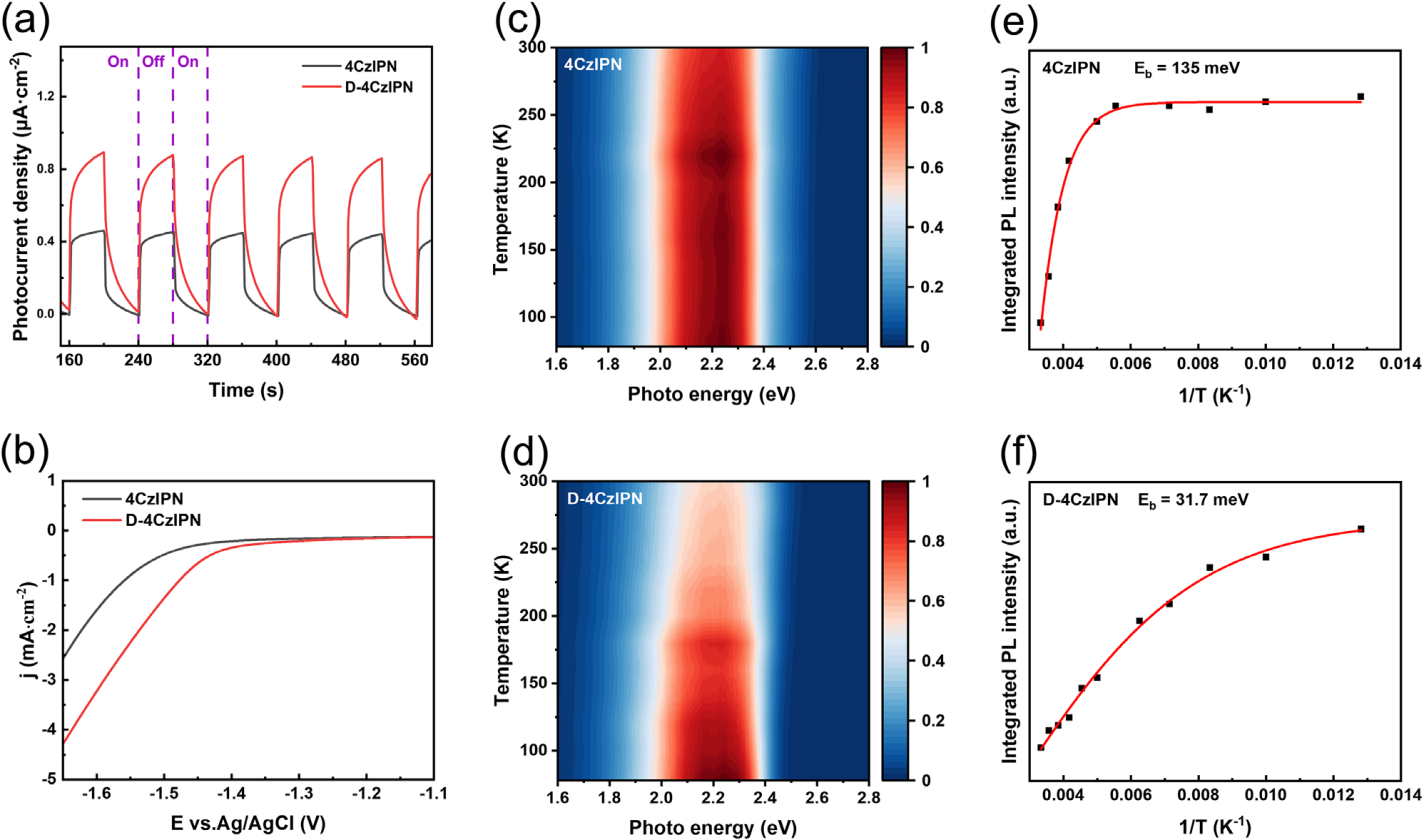
a) Chopped photocurrent density recorded under visible light illumination. b) LSV curves of 4CzIPN and D‐4CzIPN. c,d) Temperature‐dependent PL spectra of 4CzIPN and D‐4CzIPN. e,f) Integrated PL intensity of 4CzIPN and D‐4CzIPN from 78 and 300 K.

To dig out the underlying origin of the enhanced photocatalytic performance for D‐4CzIPN, the light absorption property of 4CzIPN and D‐4CzIPN was first studied. No obvious spectral differences were observed between 4CzIPN and D‐4CzIPN (Figure S8, Supporting Information), suggesting that deuteration had little influence on the energy levels involved in the radiative decay in the solution state. **Figure** **2**a compared the visible light photocurrent responses of 4CzIPN and D‐4CzIPN. It could be noticed that D‐4CzIPN showed a higher photocurrent response when the light was switched on and a slower photocurrent decay after the light was switched off, indicating that the D‐4CzIPN had a better charge separation efficiency. As shown in Figure S9 (Supporting Information), D‐4CzIPN exhibited a smaller arc radius in the EIS Nyquist plot compared to 4CzIPN, further confirming that deuteration facilitated the separation and transport of photogenerated charge carriers. The linear sweep voltammetry (LSV) curves, as shown in Figure 2b and Figure S10 (Supporting Information) also revealed an obviously higher current density of D‐4CzIPN. Furthermore, steady‐state temperature‐dependent photoluminescence spectroscopy (TD‐PL) measurements were performed at the temperature range of 78–300 K (Figure 2c–f) to investigate the separation of photogenerated charge carriers. The exciton binding energy (*E_b_*) and exciton dissociation efficiency (EDE) were estimated by fitting the integrated PL emission spectra using the Arrhenius equations:^[^ ^37^, ^38^, ^39^ ^]^

(2) $$I\left(T\right)=\frac{I_{0}}{1+Ae^{-\frac{E_{b}}{k_{B}T}}}$$

(3) $$EDE=e^{-\frac{E_{b}}{k_{B}T}}$$

where *I_0_* is the PL intensity at 0 K, *k_B_* is the Boltzmann constant, *T* is the temperature, *k_B_T* ≈25 meV at room temperature, and *E_b_* is the binding energy of an exciton. The fitting results yielded the *E_b_* of 4CzIPN and D‐4CzIPN as 135 and 31.7 meV, respectively, and the EDE for 4CzIPN and D‐4CzIPN were calculated to be 0.4% and 28.1%. These results indicated a weaker exciton binding energy and a better exciton dissociation efficiency for D‐4CzIPN.

**Figure 3:**
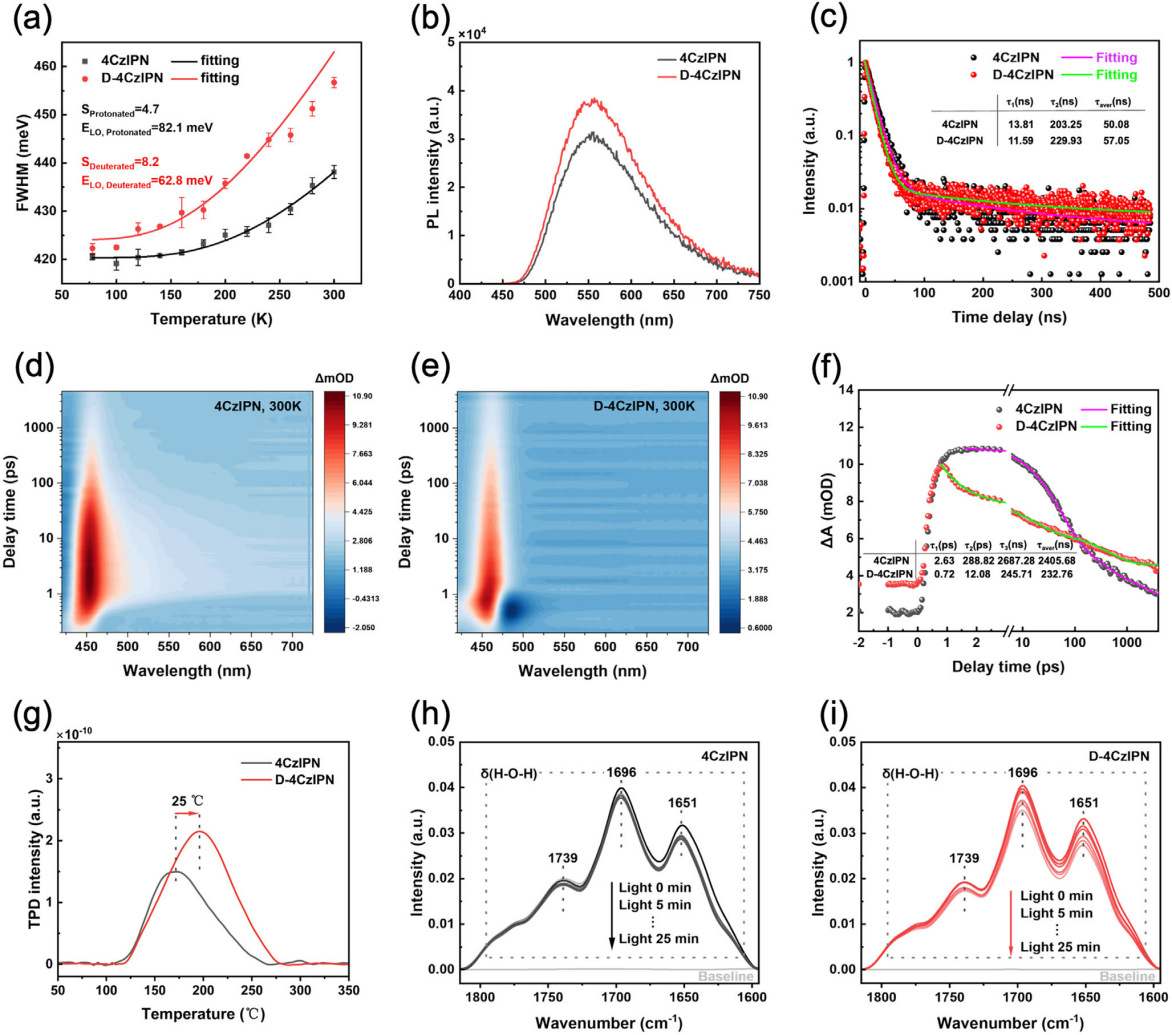
a) The Huang‐Rhys factor determined by fitting the FWHM of the steady‐state PL spectra with temperature. b) The steady‐state PL spectra of 4CzIPN and D‐4CzIPN (10‐3 mol L^−1^). c) The time‐resolved fluorescence spectra. d,e) Transient absorption spectra and f) the absorbance delay time profiles at 456 nm for 4CzIPN and D‐4CzIPN. g) H_2_O‐TPD profiles of 4CzIPN and D‐4CzIPN. The in situ FTIR spectra recorded over h) 4CzIPN and i) D‐4CzIPN as a function of irradiation time.

As the movement and decay dynamics of charge carriers could also be greatly influenced by their interaction with phonons and the nonradiative recombination, two nonradiative energy dissipation pathways were taken into consideration: 1) electron‐phonon scattering during the ultrafast cooling of hot carriers, and 2) nonradiative recombination of electron–hole pairs. First, the dependence of the rising dynamics of hot carriers on the excitation fluence was investigated in detail (Figure S11, Supporting Information). Through exponential fitting, the rising time was estimated to be on the time scale of hundreds of femtoseconds and had a linear relationship with the pump fluence, indicating the presence of electron‐phonon coupling and its crucial role on the carrier relaxation process.^[^ ^40^ ^]^ As compared to 4CzIPN (τ_e‐ph_: 90.7 fs), a shorter intrinsic electron‐phonon coupling time was obtained in D‐4CzIPN (τ_e‐ph_: 79.2 fs), implying the promotional effect of the deuteration strategy on accelerating the relaxation of hot electrons (S_n_) to the reactive excited states (S_1_). Additionally, the excess energy from the cooling of hot carriers could be efficiently transferred to the internal phonon system to thermalize the lattice.^[^ ^41^ ^]^ The elevation of the lattice temperature would facilitate exciton dissociation into free carriers, in accordance with the calculated higher exciton dissociation efficiency. Then, TD‐PL spectroscopy was further applied to investigate the nonradiative recombination of electron–hole pairs. The Huang–Rhys factor S, that revealing the electron‐phonon coupling strength, could be determined by extracting the PL linewidth at different temperatures from TD‐PL spectra using Equation (4):^[^ ^42^, ^43^ ^]^

(4) $$FWHM=2.36\sqrt{S}\hbar \omega_{phonon}\sqrt{\coth\frac{\hbar \omega_{phonon}}{2k_{B}T}}$$

where *FWHM* represents the full width at half maximum of the PL linewidth, *ω_phonon_* denotes the longitudinal optical (LO) phonon frequency, *k_B_* is the Boltzmann constant, and *T* is the temperature. The Huang–Rhys factor S is a well‐applied parameter to evaluate the strength of electron‐phonon coupling; generally the larger the value of S is, the stronger the electron‐phonon coupling.^[^ ^43^ ^]^ As elaborated in **Figure** **3**a, the S values of 4CzIPN and D‐4CzIPN were determined to be 4.7 and 8.2, respectively, indicating that deuteration would greatly promote electron‐phonon coupling. In the photocatalytic process, the internal conversion (IC) between high vibrational energy levels of the ground state (S_0_) and low vibrational energy levels of the excited state (S_1_) is the primary driver of the nonradiative recombination of electron–hole pairs. Based on Fermi's golden rule, the rate of IC is inversely correlated to the Huang–Rhys factor.^[^ ^28^ ^]^ Therefore, the enhanced electron‐phonon coupling in D‐4CzIPN indicated a suppressed rate of IC and a reduced non‐radiative decay.^[^ ^26^ ^]^ Consequently, the deuteration strategy on one hand could accelerate the relaxation of hot electrons (S_n_) to the reactive states (S_1_) for chemical reactions, while on the other hand could inhibit the nonradiative energy dissipation from S_1_ to S_0_.

To further elucidate the deuteration effect on charge carrier dynamics and energy dissipation, fluorescence and ultrafast transient absorption spectroscopy measurements were conducted on both 4CzIPN and D‐4CzIPN. As shown in the transient and steady‐state fluorescence measurement results (Figure 3b,c; Table S1, Supporting Information), D‐4CzIPN exhibited not only an increased fluorescence intensity but also an extended fluorescence lifetime (57.05 ns) relative to 4CzIPN (50.08 ns). The enhanced fluorescence intensity might be ascribed to the increased Franck–Condon factors due to reduced displacement between ground and excited‐state potential energy surfaces upon deuteration,^[^ ^26^ ^]^ and the prolonged lifetime was possibly due to the decrease in phonon frequency that might reduce the carrier migration rate and thus elongate the radiative decay process of photogenerated charges.^[^ ^44^ ^]^ As displayed in Figure 3d–f and Figure S12 (Supporting Information), a positive absorption signal at ≈456 nm could be clearly observed on 4CzIPN and D‐4CzIPN upon 360 nm excitation, which was due to the absorption of the excited states. The relatively narrower absorption band for D‐4CzIPN indicated a suppressed intersystem crossing (ISC), given the small energy gap between the singlet state (S_1_) and the triplet state (T_1_).^[^ ^14^ ^]^ The absorbance delay time profiles at 456 nm were fitted by a third exponential decay function (Figure 3f; Table S2, Supporting Information). The fastest time constant (τ_1_) likely represents the vibrational cooling of S_n_ to the relaxed S_1_ state; τ_2_ accounts for a combination of IC from S_1_ to S_0_, prompt decay of S_1_ population by fluorescence, and ISC from the lowest vibrational levels of S_1_ to the manifold of triplet states; and τ_3_ is the time constant for delayed fluorescence and IC resulting from reversed ISC. It was evident that the average carrier relaxation rate of D‐4CzIPN was much faster than that of 4CzIPN, indicating an accelerated decay from the excited state to ground state in D‐4CzIPN. Since both the fluorescence intensity and fluorescence lifetime of D‐4CzIPN were enhanced, along with suppressed ISC, the accelerated decay further demonstrated an effective mitigation of non‐radiative energy dissipation by deuteration.

Apart from carrier dynamics, the interaction between the deuterated photocatalyst and water was also investigated by H_2_O temperature‐programmed desorption (H_2_O‐TPD). Compared to 4CzIPN, D‐4CzIPN showed a higher water desorption temperature (Figure 3g), indicating its stronger interaction with H_2_O,^[^ ^45^ ^]^ which would expect to benefit photocatalytic hydrogen evolution.^[^ ^46^ ^]^ Additionally, as shown in Figure 3h,i, the in situ FTIR spectra acquired as a function of irradiation time displayed three bands at 1651, 1696, and 1739 cm^−1^, corresponding to the bending modes of three‐coordinated surface water with dangling oxygen, four‐coordinated surface water, and three‐coordinated surface water with dangling hydrogen, respectively.^[^ ^47^ ^]^ It could be found that the intensity of the water bending modes on D‐4CzIPN decreased more significantly than that on 4CzIPN, indicating enhanced water dissociation on the D‐4CzIPN surface under light illumination.

**Figure 4:**
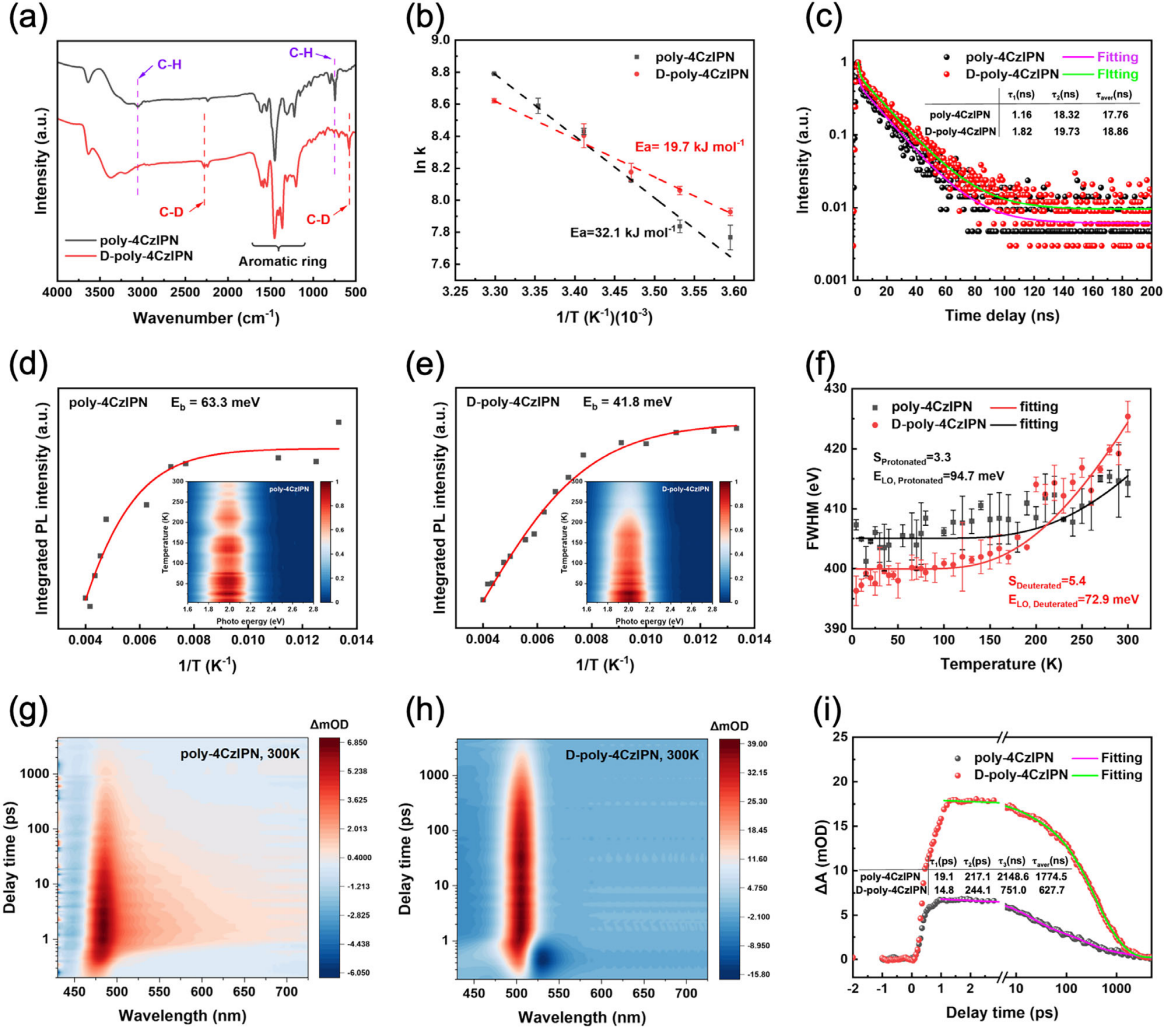
a) The FTIR spectra. b) Arrhenius diagram of poly‐4CzIPN and D‐poly‐4CzIPN in the temperature range of 5–30 °C. c) The time‐resolved fluorescence spectra. d,e) Temperature‐dependent PL spectra and integrated PL intensity for poly‐4CzIPN and D‐poly‐4CzIPN from 78 to 300 K. f) The Huang–Rhys factor (S) by fitting the FWHM of the steady‐state PL spectra with temperature. g,h) Transient absorption spectra and i) the absorbance delay time profiles at 456 nm for poly‐4CzIPN and D‐poly‐4CzIPN.

To assess the universality of the deuteration strategy, the high vibration frequency C─H bonds in poly‐4CzIPN were substituted by D to form heavier C─D bonds (D‐poly‐4CzIPN). As expected, XRD and XPS analyses (Figures S13 and S14, Supporting Information) revealed no significant changes in structures and chemical valence states between poly‐4CzIPN and D‐poly‐4CzIPN. It was interesting to note that D‐poly‐4CzIPN displayed a decreased light absorption ability (Figure S15, Supporting Information), of which the bandgap was determined to be 1.77 eV, close to that of poly‐4CzIPN (1.75 eV). As displayed in the FTIR spectra (**Figure** **4**a), D‐poly‐4CzIPN also showed reduced stretching and bending energies of the C─H bonds. The stretching vibration energy of the C─H bond decreased by a factor of $\sqrt{2}$ after deuteration, demonstrating successful substitution of H by D in D‐poly‐4CzIPN. Similar deuteration effect on photocatalytic activity was also observed in the polymerized photocatalysts (Figure S16, Supporting Information), which also presented outstanding structure and activity stability (Figures S17 and S18, Supporting Information). As shown in the Arrhenius diagram (Figure 4b), the D‐poly‐4CzIPN possessed a lower activation energy of 19.7 kJ mol^−1^ as compared with that of poly‐4CzIPN (32.1 kJ mol^−1^). To confirm the mechanism behind, the electron‐phonon coupling strength and charge carrier dynamics of poly‐4CzIPN and D‐poly‐4CzIPN were evaluated in detail. The D‐poly‐4CzIPN also exhibited an increased fluorescence intensity (Figure S19, Supporting Information) and an extended fluorescence lifetime (Figure 4c; Table S3, Supporting Information), indicating deuteration‐induced suppression of non‐radiative decay and promotion on radiative decay of photogenerated charge carriers. As shown in Figure 4d,e, the exciton binding energy of D‐poly‐4CzIPN was determined to be 41.8 meV, which was lower than that of poly‐4CzIPN, revealing the promoted exciton dissociation ability of D‐poly‐4CzIPN. The fitted S factor (Figure 4f) indicated that the electron‐phonon coupling strength of D‐poly‐4CzIPN (S = 5.4) was stronger than that of poly‐4CzIPN (S = 3.3). Besides, TAS measurements (Figure 4g–i; Figure S20 and Table S4, Supporting Information) noticed similar phenomena of a narrowed absorption band and accelerated carrier decay in D‐poly‐4CzIPN. In combination with the extended fluorescence lifetime and increased fluorescence intensity, the inhibition of non‐radiative decay by deuteration was demonstrated. Additionally, the higher water desorption temperature (Figure S21, Supporting Information) and faster decrease of water bending intensity (Figure S22, Supporting Information) were also observed on D‐poly‐4CzIPN, further demonstrating a universal deuteration strategy for boosting photocatalytic hydrogen evolution efficiency.

## 3 Conclusion

In summary, a deuteration strategy has been successfully developed to promote photocatalytic hydrogen production efficiency. The deuterated molecular and polymerized photocatalysts (D‐4CzIPN and D‐poly‐4CzIPN) are synthesized by replacing high‐energy C─H bonds with heavier C─D bonds. Comprehensive characterizations show that the deuterated photocatalysts possess lower and denser packing of vibrational energy levels and stronger electron‐phonon coupling, contributing to more efficient exciton dissociation and inhibition of energy dissipation, greatly promoting the photocatalytic hydrogen evolution reaction. Our findings here provide new perspectives and insights for designing high‐efficiency photocatalysts via tailoring the electron‐phonon coupling strength.

## Conflict of Interest

The authors declare no conflict of interest.

## Author Contributions

H.S., Z.Z., Y.M., and Q.X. contributed equally to this work. H.S. contributed to writing (review and editing), funding acquisition, formal analysis, and data curation. Z.Z. contributed to writing (original draft), investigation, formal analysis, and data curation. Y.M., Q.X., Y.L., C.H., Y.Z., X.C., Y.W., and Z.F. contributed to formal analysis and data curation. J.Z. contributed to writing (review and editing), formal analysis, and data curation. C.S., F.F., and B.L. contributed to supervision, funding acquisition, and formal analysis.

## Supporting information



Supporting Information

## Data Availability

The data that support the findings of this study are available from the corresponding author upon reasonable request.
