# Supplementary material for: Deuteration Induced Electron‐Phonon Coupling Modulation: Suppressing Energy Dissipation and Enhancing Carrier Separation in Organic Photocatalysis
Source: Adv Sci (Weinh). 2025 Nov 19;13(7):e16740. doi: 10.1002/advs.202516740 (PMC12866818; doi:10.1002/advs.202516740)
Supplement: Supplementary file 1 — Supporting Information [file ADVS-13-e16740-s001.docx]

**Supporting Information**

**Deuteration induced electron-phonon coupling modulation: suppressing energy dissipation and enhancing carrier separation in organic photocatalysis**

*Hongli Sun^†^,* *Zirui Zhou^†^,* *Yunfei Ma^†^,* *Qingzhu Xu^†^,* *Yanglong Liao, Cheng Han, Yue Zheng,* *Xiaosong Cao,* *Yu Wang,* *Zutao Fan, Jianfeng Zhao,* *Chenliang Su^*^,* *Fengtao Fan^*^ and* *Bin Liu^*^*

[*] Prof. H. Sun^†^, Dr. Z. Zhou^†^, Dr. Y. Ma ^†^, Dr. Q. Xu ^†^, Dr. Y. Liao, Prof. C. Han, Prof. Y. Zheng, Prof. Y. Wang, Dr. Z. Fan, Prof. C. Su^*^

International Collaboration Laboratory of 2D Materials for Optoelectronics Science and Technology of Ministry of Education, Institute of Microscale Optoelectronics, Shenzhen University, Shenzhen 518060, China

E-mail: [chmsuc@szu.edu.cn](mailto:chmsuc@szu.edu.cn)

Prof. H. Sun^†^, Dr. Z. Zhou^†^, Dr. Y. Ma^†^, Dr. Y. Liao, Prof. C. Han, Prof. Y. Zheng, Prof. C. Su^*^

State Key Laboratory of Radio Frequency Heterogeneous Integration, Shenzhen University, Shenzhen 518060, China

Dr. Q. Xu^†^

College of Chemistry and Chemical Engineering, Yangzhou University, Yangzhou 225002, China

Prof. X. Cao

Shenzhen Key Laboratory of New Information Display and Storage Materials, College of Materials Science and Engineering, Shenzhen University, Shenzhen 518060, China

Prof. J. Zhao, Prof. F. Fan^*^

State Key Laboratory of Catalysis, Dalian National Laboratory for Clean Energy, The Collaborative Innovation Centre of Chemistry for Energy Materials (iChEM), Dalian Institute of Chemical Physics, Chinese Academy of Sciences, Dalian, 116023, China

E-mail: ftfan@dicp.ac.cn

Prof. B. Liu^*^

Department of Materials Science and Engineering, City University of Hong Kong, Hong Kong SAR 999077, China

Department of Chemistry, Hong Kong Institute for Clean Energy (HKICE) & Center of Super-Diamond and Advanced Films (COSDAF), City University of Hong Kong, Hong Kong SAR 999077, China

E-mail: bliu48@cityu.edu.hk

[^+^] These authors contributed equally to this work.

**Experimental section**

**Materials**

All the reagents were of analytical grade and were used as received. All aqueous solutions were prepared using deionized water. Carbazole, carbazole-D8 (98%D，99%), and tetrafluoroisophthalonitrile were purchased from Adamas-beta. Ferric chloride, H_2_PtCl_6_.6H_2_O (AR, Pt ≥ 37.5%) and triethanolamine (TEOA, ≥ 99.0%) were purchased from Aladdin Reagent (Shanghai, China). NaH was purchased from ACMEC (Shanghai, China). Hydrochloric acid (HCl, 36%), N, N-dimethylformamide (DMF), tetrahydrofuran (THF) and methanol were obtained from Sinopharm Chemical Reagent Co., Ltd. (Shanghai, China).

**Sample preparation**

***Preparation of 4CzIPN/D-4CzIPN:*** Under an argon atmosphere, a solution of carbazole (875 mg, 5.0 mmol)/carbazole-*D*8 (880 mg, 98%D, 5.0 mmol) in anhydrous THF (20 mL) was slowly added to a NaH solution (60% oil suspension, 600 mg, 15 mmol) in anhydrous THF (20 mL) under rigorous stirring. After 30 minutes, tetrafluoroisophthalonitrile (200 mg, 1.0 mmol) was added. The reaction mixture was stirred at room temperature for 12 hours, followed by adding 2 mL of water to quench the excess NaH. The resulting mixture was concentrated under reduced pressure and washed with water and ethanol. The crude product was obtained and further purified by recrystallization from hexane/CH_2_Cl_2_ or acetone/CHCl_3_, followed by silica gel column chromatography (CH_2_Cl_2_: hexane, 4:1 v/v), yielding the corresponding monomer product 4CzIPN (725 mg, yield: 92%), D-4CzIPN (765 mg, yield: 93%, 98%D).

***Preparation of poly-4CzIPN/poly-D-4CzIPN*:** The monomer (500 mg) was first dissolved in anhydrous CHCl_3_ (50 mL) under an argon atmosphere. The monomer solution was then added to a mixture of anhydrous FeCl_3_ (644 mg, 4.0 mmol) and anhydrous CHCl_3_ (100 mL), and stirred at room temperature for 72 hours. Afterwards, methanol (50 mL) was added and stirred for 1 hour to quench the reaction. The mixture was then washed with 12 M HCl for 1 hour, followed by concentrated under reduced pressure, and washed with deionized water. The resulting product was further purified by Soxhlet extraction first with THF for 24 hours, and then with methanol for 48 hours. The product was dried under vacuum at 80 °C for 24 hours to obtain the corresponding polymer (473 mg, yield: 94%).

**Characterizations**

X-ray diffraction (XRD) analysis was conducted using Cu Kα radiation (Rigaku Ultima IV) in the 2θ range of 10° to 80° at a scan rate of 5° min^-1^, a current of 40 mA and a voltage of 40 kV. UV-vis-DRS spectra were recorded on a Varian Cary 5000 Scan UV–visible system (Agilent), with 100% BaSO_4_ as the internal standard. Fourier-transform infrared spectroscopy (FTIR) measurements were carried out on a VERTEX 70 V spectrometer (Bruker), with KBr as the test background in the wavenumber range of 40-4000 cm^-1^. H_2_O temperature-programmed desorption (H_2_O-TPD) was conducted on a CATLAB spectrometer (Hiden Analytical). Photoluminescence (PL) and time-resolved transient photoluminescence (TR-PL) measurements were performed on a HORIBA Fluorolog-3 spectrofluorometer. Temperature-dependent photoluminescence (TD-PL) measurements were carried out on a Scan Pro Advance photoelectric testing system (Metatest Corporation). X-ray photoelectron spectroscopy (XPS) spectra were recorded on a Thermo Scientific K-Alpha spectrometer equipped with a monochromatic Al Kα X-ray source. All electrochemical measurements were conducted in a three-electrode quartz cell controlled by an electrochemical workstation (CHI660E). A Pt plate and Ag/AgCl electrode were used as the counter electrode (CE) and reference electrode (RE), respectively. The working electrode was prepared on fluorine-doped tin oxide (ITO) glass with an exposed area of 1 cm^2^. Transient photocurrent response curves were recorded under visible light irradiation (λ ≥ 420 nm) using a 300 W Xenon lamp source (PLS-SXE 300D, Beijing Perfectlight Technology Co., Ltd.). Linear sweep voltammetry (LSV) measurements were carried out in an electrolyte of 2 M Na_2_SO_4_ solution. Transient absorption (TA) spectroscopy was carried out using an optical instrument combined with a frequency-doubled mode-locked Ti:sapphire femtosecond laser (coherent) and an optical parametric amplifier (OPA) system. NMR spectra were recorded on a Bruker AV 600 MHz spectrometer at 25 ºC in the solvents indicated. Chemical shifts (*δ*) are reported in ppm and respectively referenced to internal standard Me_4_Si and solvent signals. HRMS spectra were recorded on a solariX 70-FT-MS.

**Photocatalytic measurements**

The photocatalytic hydrogen evolution experiments were performed in a Labsolar-6A all-glass automatic online micro gas analysis system (Perfectlight). Chloroplatinic acid hexahydrate was used as the Pt precursor. Prior to the reaction, 10 mg of catalyst and Pt precursor (3 wt.% relative to the catalyst) were added into a double-layer quartz photoreactor containing 50 mL of triethanolamine (TA) aqueous solution (10 vol.%). The mixture was then evacuated, and Pt was photo-deposited under vacuum for 10 minutes. After photodeposition, the photocatalytic reaction was carried out under vacuum at 10 °C, using a 300 W Xe lamp (PLS-SXE 300D, Perfectlight) with a UV cutoff filter (λ ≥ 420 nm). The evolved gases were analyzed online by gas chromatography (GC9790 Plus, Fuli Instruments) equipped with a thermal conductivity detector and a Fuli 5A molecular sieve column, using argon as the carrier gas.

**Time-resolved transient photoluminescence (TR-PL) decay spectra:**

The transient photoluminescence (TR-PL) decay curves were fitted using a tri-exponential model:

$$I\left( t \right)=I_{0}+A_{1}\exp\left( -\frac{t}{\tau_{1}} \right)+A_{2}\exp\left( -\frac{t}{\tau_{2}} \right)+A_{3}\exp\left( -\frac{t}{\tau_{3}} \right)$$

$$\tau_{n}=\frac{A_{1}\tau_{1}^{2}+A_{2}\tau_{2}^{2}+A_{3}\tau_{3}^{2}}{A_{1}\tau_{1}+A_{2}\tau_{2}+A_{3}\tau_{3}}$$

where I_0_ represents the baseline correction value, and A_1_, A_2_, and A_3_ denote the amplitudes corresponding to the fluorescence lifetimes τ_1_, τ_2_, and τ_3_, respectively.

**State temperature-dependent photoluminescence (TD-PL) spectroscopy**

Steady-state temperature-dependent photoluminescence (TD-PL) measurements were carried out on a Scan Pro Advance photoelectric testing system (Metatest Corporation). A pellet was prepared by compressing 100 mg of the sample. This pellet was placed in the home-made vacuum chamber, of which the pressure was evacuated to 10^-5^ mbar. The pellet was maintained in this chamber for 3 hours. Subsequently, liquid helium was introduced into the system. After the temperature was kept constant for 30 min, the photocatalyst was then excited using a 405 nm laser, and its TD-PL spectra were recorded over a temperature range of 10–300 K.

**Temperature-programmed desorption measurements (****H_2_O TPD)**

A 10 mg catalyst sample was loaded into a 1/8-inch quartz tube, which was then placed into the sample chamber of the TPD apparatus. A carrier gas flow of Ar at 50 mL/min was introduced into the system. The sample was first pretreated at 260 °C under vacuum for 2 hours. After the furnace temperature cooled to 50 °C, water vapor was introduced into the TPD chamber for 10 min using Ar as the carrier gas. The sample was then heated to 100 °C and held for 1 hour to remove any physiosorbed water introduced during handling. Then the temperature of the sample cooled down to 50°C. Finally, a temperature-programmed heating process from 50°C to 400°C was applied, during which the signals of chemically adsorbed water desorbed from the sample surface were detected in real time using mass spectrometry.

***In-situ* FTIR measurements of water splitting**

*In-situ* FTIR spectra were recorded on a VERTEX 70 V spectrometer (Bruker), with KBr as the test background in the wavenumber range of 40-4000 cm^-1^. A transparent wafer was prepared by thoroughly grinding 2 mg of the catalyst with 300 mg of KBr. This wafer was placed in the sample chamber, and a background spectrum was collected under vacuum and automatically subtracted. Water vapor was then introduced into the chamber by an Ar flow (50 mL/min) for a 30-minute dark adsorption period. Finally, the sample was irradiated by an internal LED light source (λ = 420 nm), and the FTIR spectra were collected simultaneously.

**Isotope-labeling test**

Two groups of isotopic deuterium labeling experiments with and without triethanolamine (TA) were conducted. For the isotopic deuterium labeling experiments without TA, 10 mg of photocatalyst loaded with 3 wt.% Pt was introduced into a double-layer quartz reactor containing 50 mL of D_2_O or H_2_O. The photocatalytic hydrogen or deuterium evolution reaction was then carried out under an argon atmosphere. After 3 hours of irradiation (λ ≥ 420 nm), the light source was turned off. The gaseous products were subsequently analyzed using a Hiden Analytical QGA mass spectrometry system. For the experiments with TA, only the solvent was replaced by 50 mL of TA aqueous solution (10 vol.%), while all other procedures remained the same.

**Experimental procedure for determining excitation-power-independent intrinsic time constants**

***Sample preparation***: An appropriate amount of deuterated (D-4-CzIPN) and non-deuterated (4-CzIPN) monomer samples were placed into separate clean 10-mL glass vials. To each vial, 4 mL of chromatographically pure DMF was added, followed by 5-minute ultrasonication to prepare homogeneous solutions (denoted as D-4-CzIPN-DMF and 4-CzIPN-DMF, respectively).

***Sample degassing and loading***: One milliliter of each solution was transferred to a micro cuvette (dimensions: 4 cm height × 1 cm width× 2 mm thickness) designed for femtosecond transient absorption spectroscopy. The cuvette was sealed with parafilm, and a vent was created using a needle puncture. High-purity argon gas was continuously purged through the solution for 30 minutes to remove dissolved oxygen, after which the vent was immediately sealed.

***Laser excitation and data acquisition***: Samples were photoexcited using a monochromatic 360-nm laser with pulse energies ranging from 0.7 μJ/pulse to 3.2 μJ/pulse. Transient absorption spectra were recorded for each excitation power.

***Kinetic analysis***: The formation kinetics were analyzed at three wavelengths: 845 nm, 855 nm, and 865 nm. The shortest formation time constant (τ₁) was extracted from triexponential fitting at each wavelength. The average τ₁ value across these three wavelengths was defined as the average apparent time constant for the corresponding excitation power.

***Plotting and intrinsic time constant extraction***: Apparent τ₁ values were plotted against laser power, generating the curves shown in Figure S3. The same fitting protocol was applied to both deuterated and non-deuterated samples. The intrinsic time constants (excitation-power-independent) were determined from the intercepts of the linear regression curves with the vertical axis (i.e., the apparent τ₁-axis): Non-deuterated sample: 90.7 fs; Deuterated sample: 79.2 fs.

**Scheme S1**. The synthesis equation of 4CzIPN monomers and D-4CzIPN monomers.


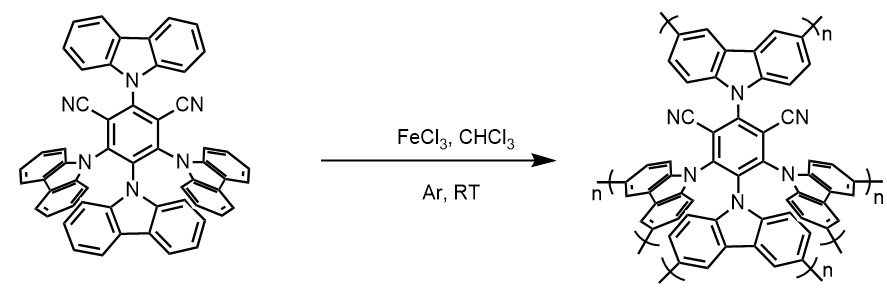


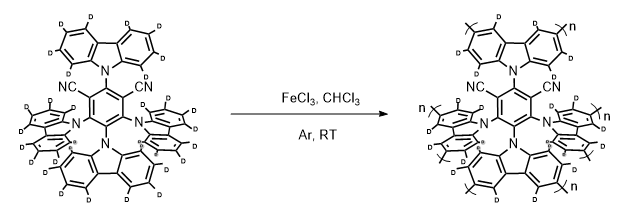


**Scheme S2.** The synthesis equation of poly-4CzIPN and D-poly-4CzIPN.

1. The XPS C1s spectra and N1s spectra of 4CzIPN and D-4CzIPN.

**
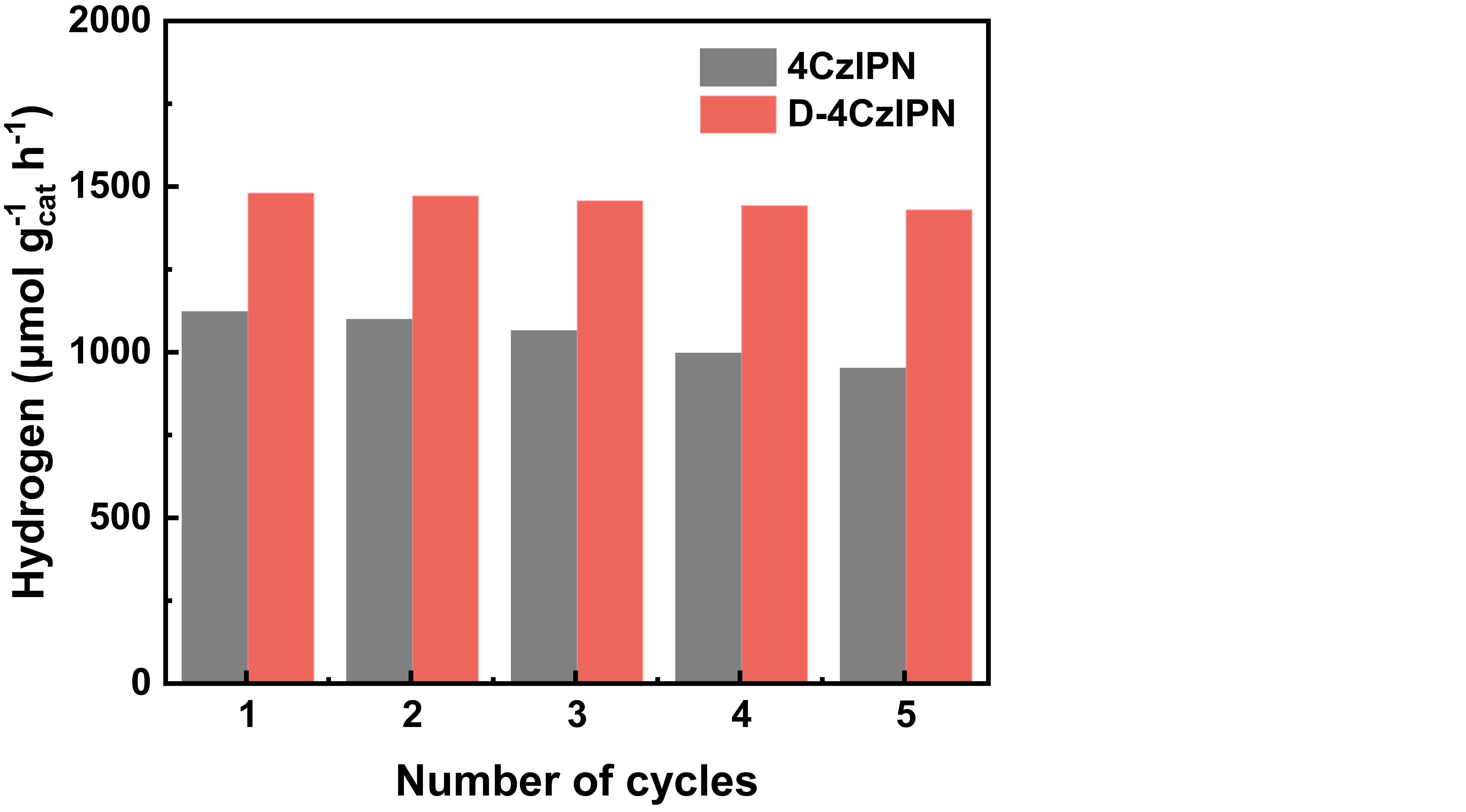
**

**Figure S2.** Photocatalytic hydrogen evolution cycling stability tests of 4CzIPN and D-4CzIPN. Reaction conditions: 10 mg catalyst, 50 mL of 10% TA aqueous solution, 3 wt.% Pt, λ ≥ 420 nm, 10 °C.

**
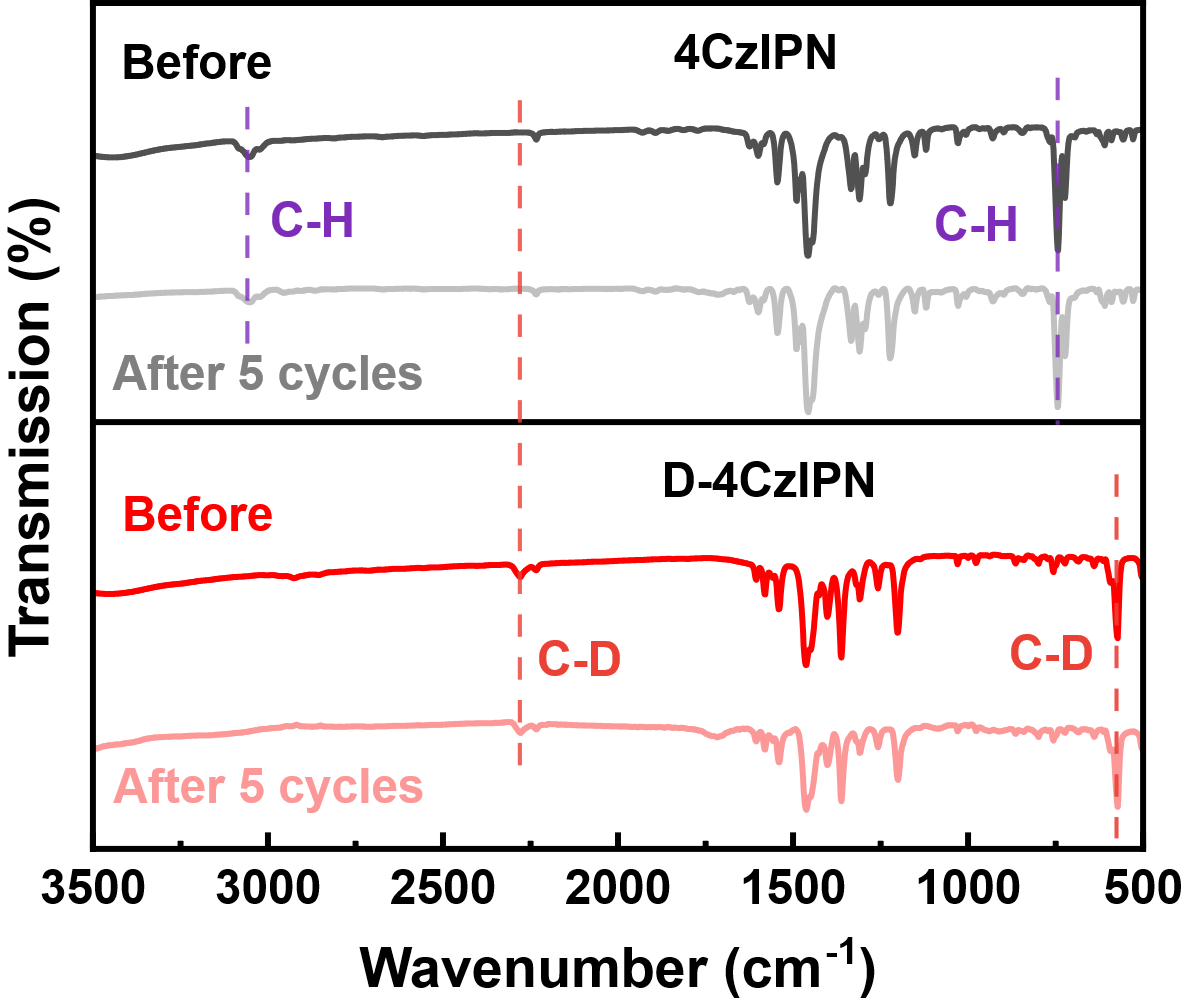
****Figure S3.** FTIR spectra of 4CzIPN and D-4CzIPN before and after 5 cycles of photocatalytic hydrogen evolution.

**
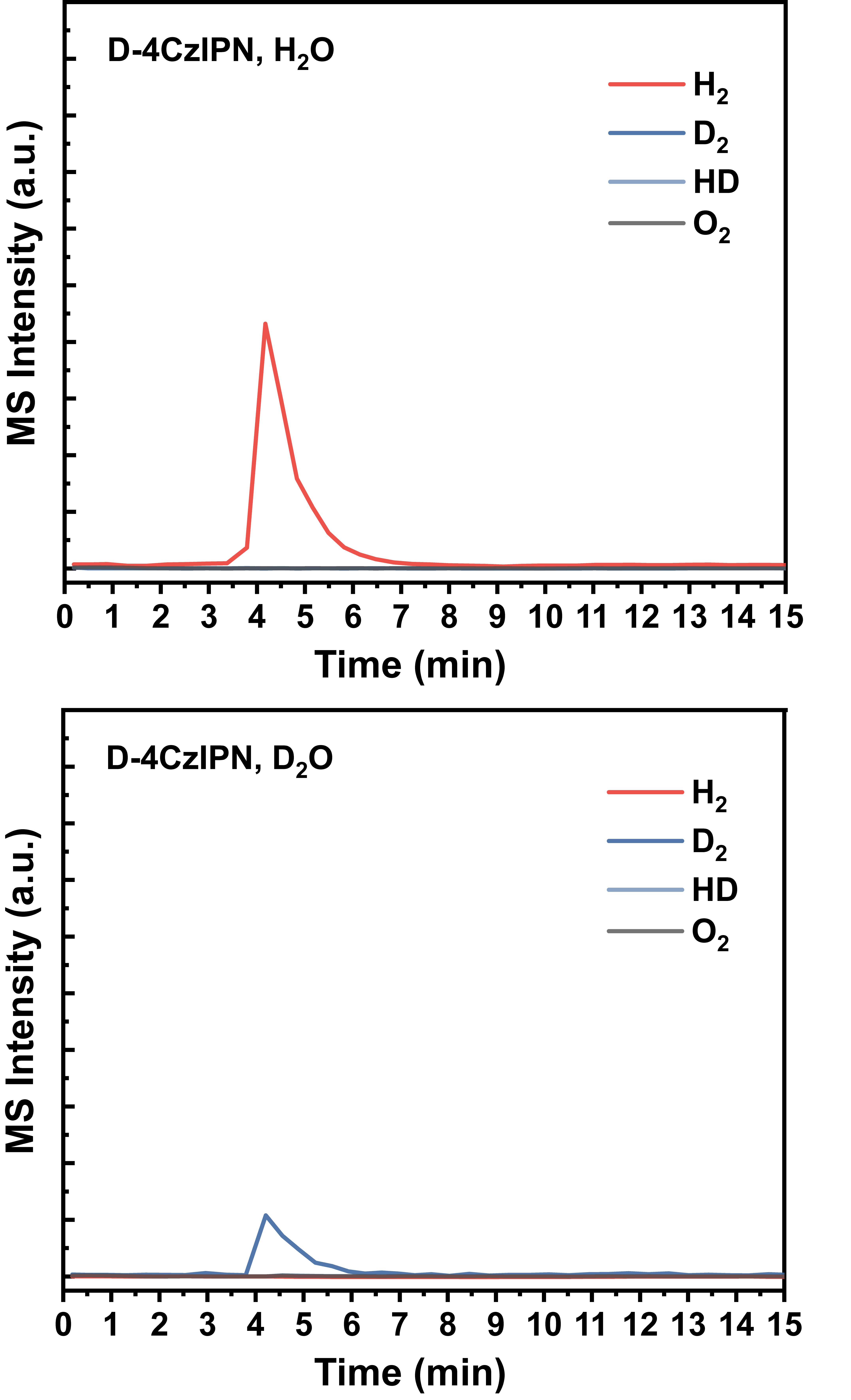
Figure S4.** Isotope labeling experiments involving D-4CzIPN were conducted under the following conditions: 10 mg of catalyst (loaded with 3 wt.% Pt), 50 mL H₂O (D₂O), λ ≥ 420 nm, and a reaction duration of 3 hours.

**
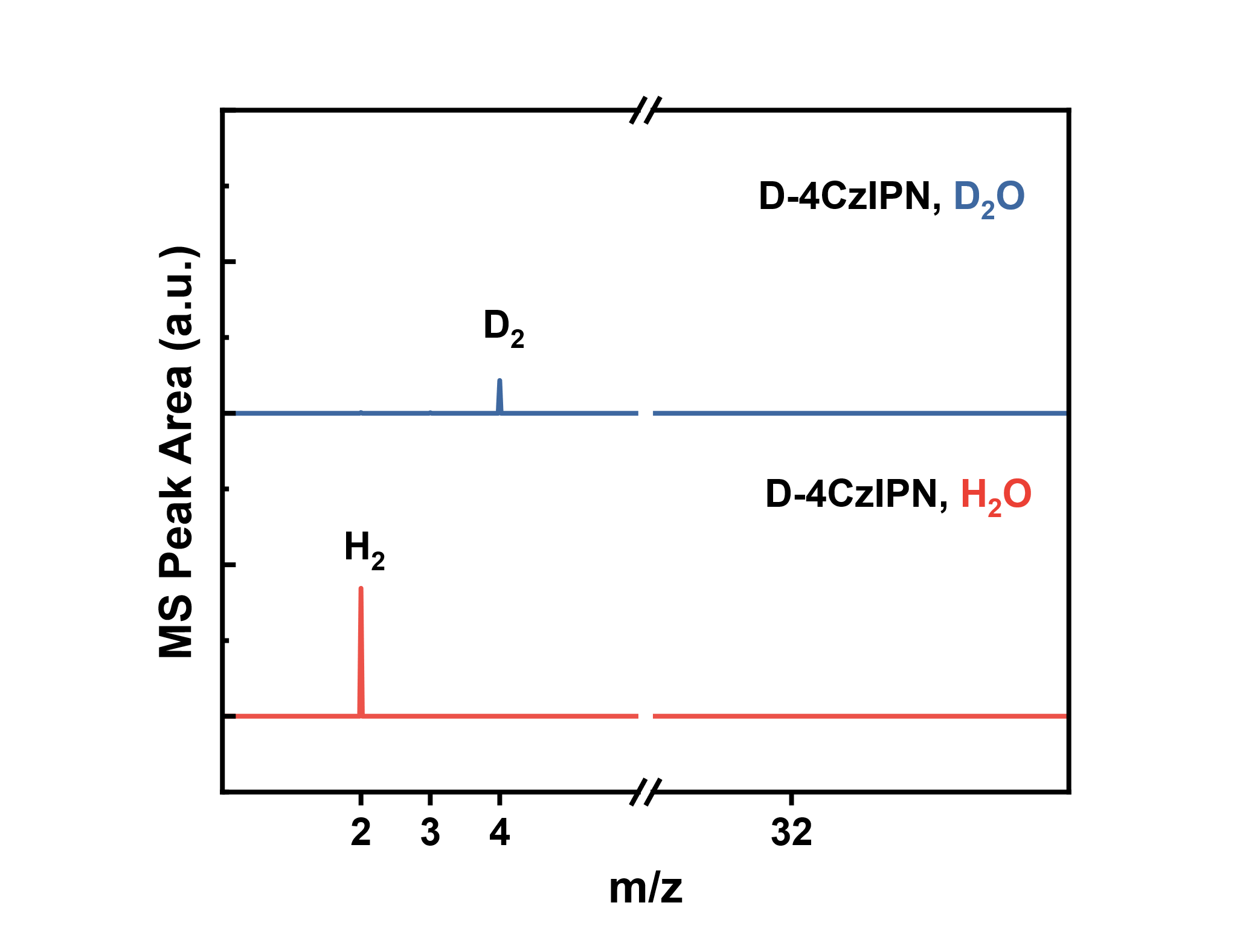
**

**Figure S5.** Comparison of mass spectrometry signals for “D-4CzIPN, D₂O” and “D-4CzIPN, H₂O”.

**
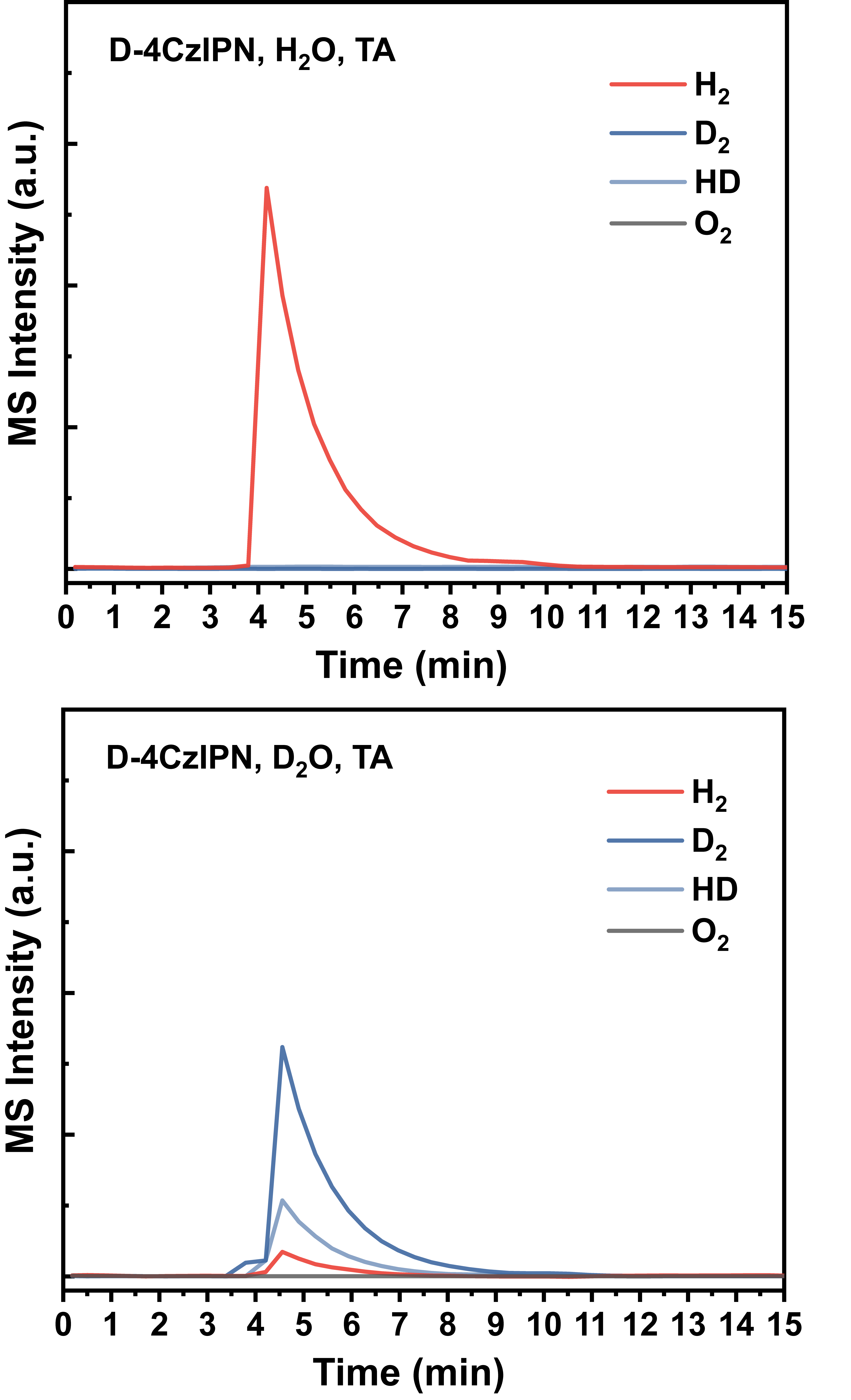
Figure S6.** Isotope labeling experiments involving D-4CzIPN were conducted under the following conditions: 10 mg of catalyst (loaded with 3 wt.% Pt), 50 mL 10 vol.% aqueous solution of TA (using either H₂O or D₂O as the solvent), λ ≥ 420 nm, and a reaction duration of 3 hours.

**

Figure S7.** Comparison of mass spectrometry signals for “D-4CzIPN, H₂O, TA” versus “D-4CzIPN, D₂O, TA”.

**Figure S8.** UV-vis spectra of 4CzIPN and D-4CzIPN in DMF solution (10^-5^ mol/L).

**

**

**Figure S9.** EIS Nyquist plots of 4CzIPN and D-4CzIPN. Measurements were conducted using a three-electrode system in a 2 M Na₂SO₄ aqueous solution under argon atmosphere.

**

**

**Figure S10.** LSV curves of 4CzIPN and D-4CzIPN with 3 wt.% Pt. Measurements were conducted using a three-electrode system in a 2 M Na₂SO₄ aqueous solution under argon atmosphere.


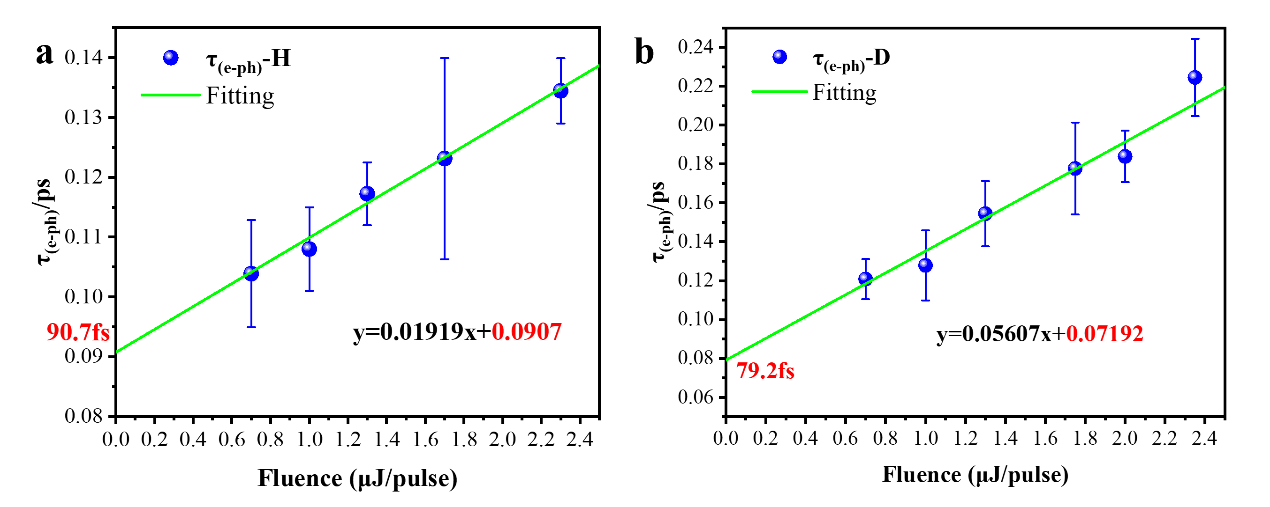


**Figure S11.** The fitted rising time constants as a function of pump fluence for the monomer of 4-CzIPN (a) and D-4-CzIPN (b) in DMF solution.

**Figure S12.** Femtosecond transient absorption measurements.

**Figure S13.** The XRD patterns of poly-4CzIPN and D-poly-4CzIPN.

**Figure S14.** The XPS C1s and N1s spectra of poly-4CzIPN and D-poly-4CzIPN.

**Figure S15.** UV-vis absorption and the Tauc plots of poly-4CzIPN and D-poly-4CzIPN.

**Figure S16.** The hydrogen production rate of poly-4CzIPN and D-poly-4CzIPN tested in the temperature range of 5 °C to 30 °C.

**
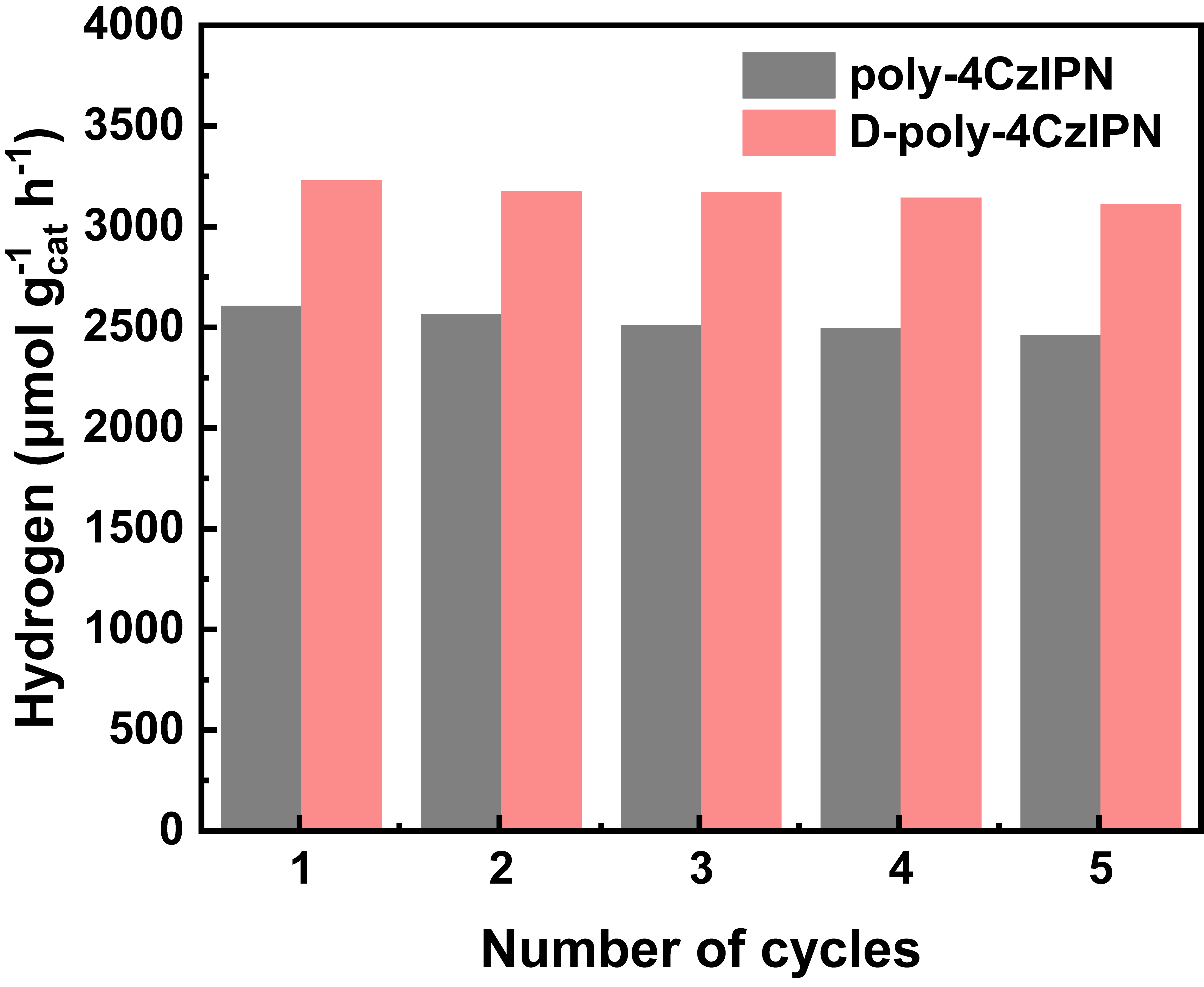
Figure S17.** Photocatalytic hydrogen evolution cycling stability tests of poly-4CzIPN and D-poly-4CzIPN. Reaction conditions: 10 mg catalyst, 50 mL of 10% TA aqueous solution, 3 wt.% Pt, λ ≥ 420 nm, 10 °C.

**Figure S18**. FTIR spectra of poly-4CzIPN and D-poly-4CzIPN before and after 5 cycles of photocatalytic hydrogen evolution.
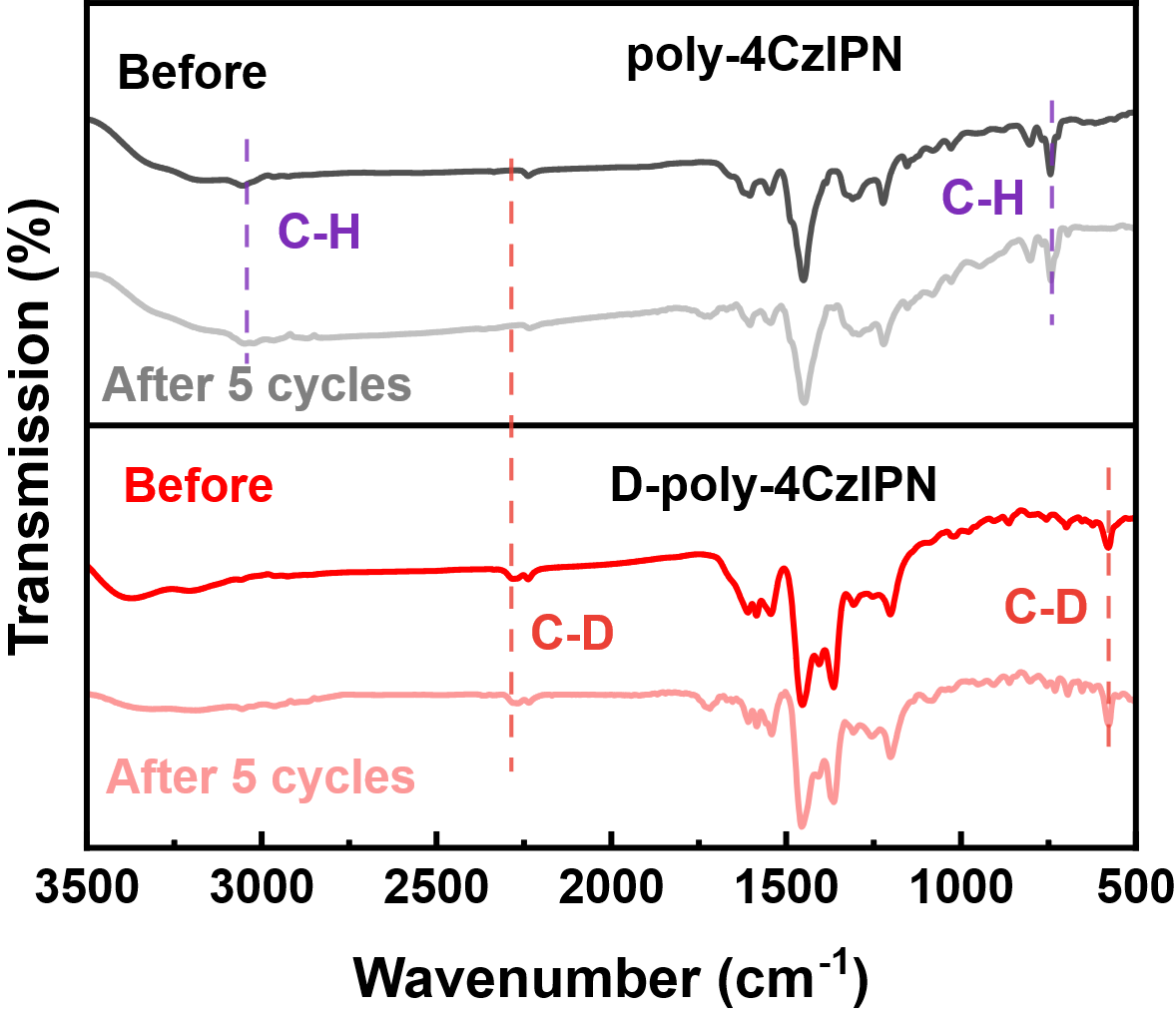

**Figure S19**. The PL spectra of poly-4CzIPN and D-poly-4CzIPN.

**Figure S20**. Femtosecond transient absorption measurements.

**
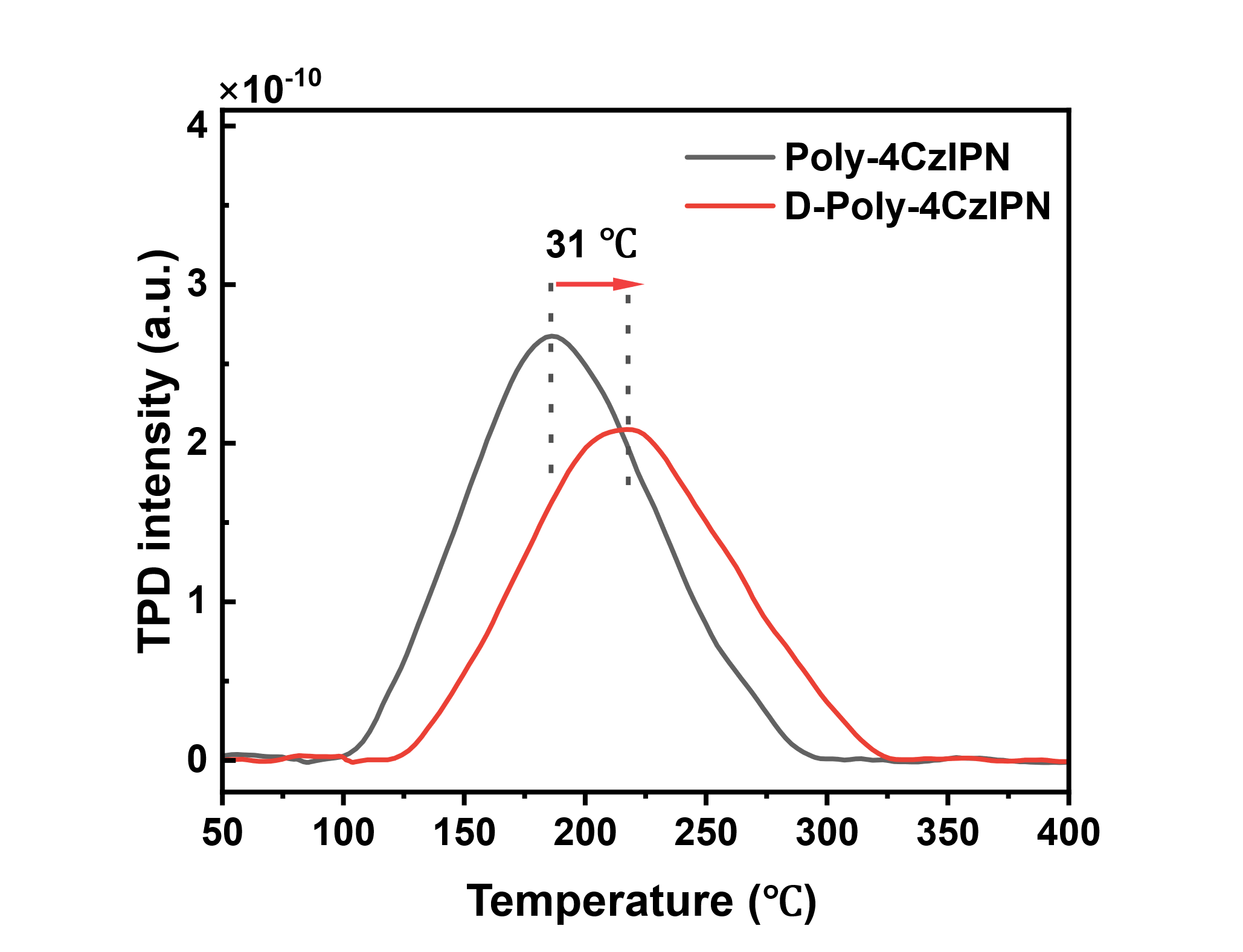
**

**Figure S21.** H_2_O-TPD profiles of poly-4CzIPN and D-poly-4CzIPN.


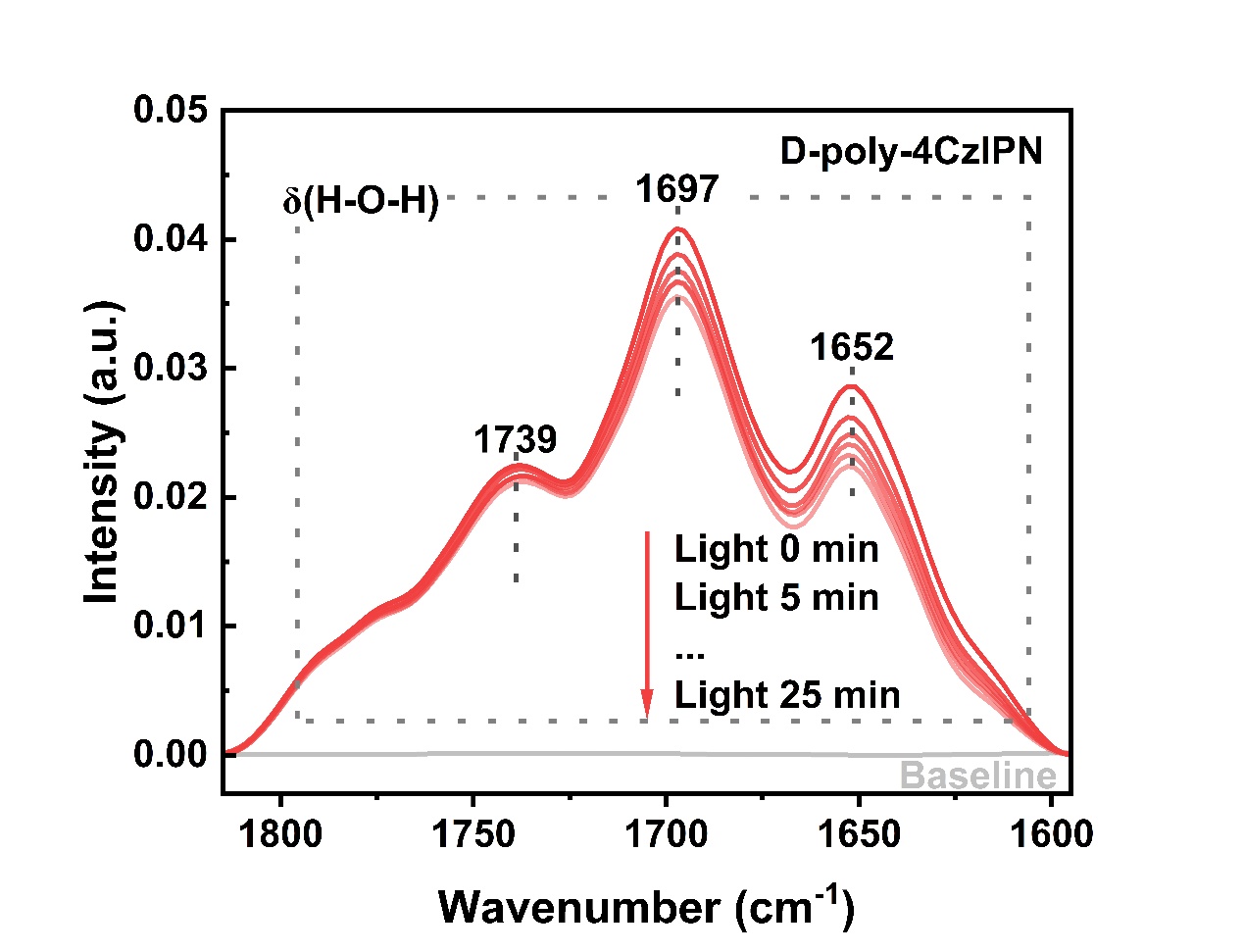


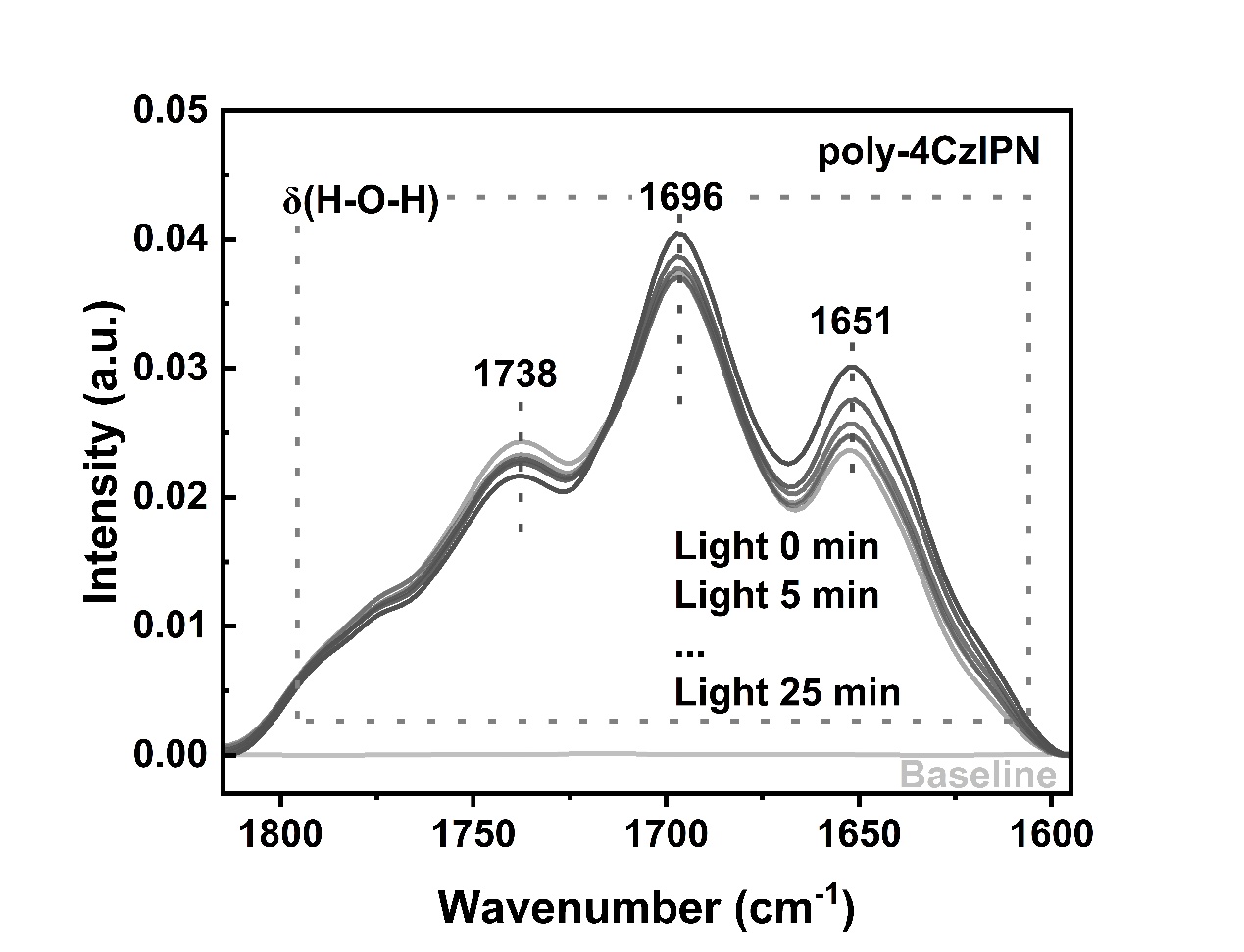


**Figure S22.** The in-situ FTIR spectra of water on the surface of D-poly-4CzIPN and poly-4CzIPN as a function of irradiation time.



**Figure S23.** Photocatalytic H₂O₂ production experiments for D-4CzIPN and 4CzIPN. Reaction conditions: 10 mg catalyst, 20 mL of 10% isopropyl alcohol (IPA) aqueous solution, O₂-saturated, λ ≥ 420 nm, 10 °C.

**Note:** “The deuterated D-4CzIPN showed a discernible improvement over 4CzIPN on photocatalytic H₂O₂ synthesis performance. This result suggests that the deuteration strategy may possess a certain generality for enhancing photocatalytic performance.”

By precisely blending protonated and deuterated carbazole precursors at varying ratios, we further prepared two deuterated 4CzIPN samples with different deuteration levels. The obtained samples were denoted as D-4CzIPN(X), where X is the percentage of deuterated carbazole precursors used in the synthesis. The performances of D-4CzIPN(0), D-4CzIPN(50), D-4CzIPN(80) and D-4CzIPN(100) in both photocatalytic hydrogen evolution (**Figure S24**) and hydrogen peroxide production (**Figure S25**) were evaluated in detail. As shown in the figures, a higher deuteration degree led to a better catalytic performance, demonstrating the positive influence of deuteration on photocatalytic performance.

**
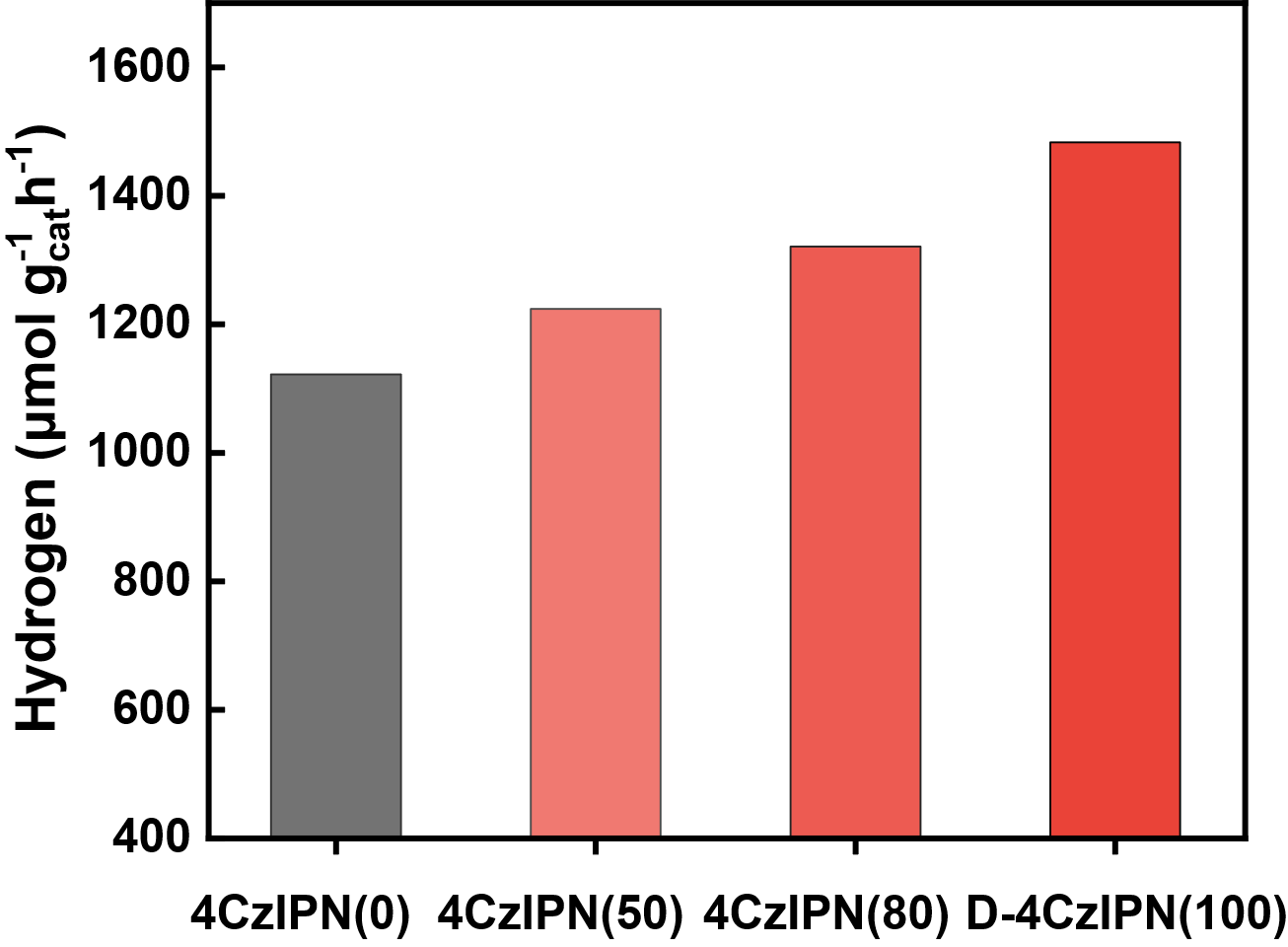
Figure S24.** Photocatalytic hydrogen evolution tests of 4CzIPN(0), 4CzIPN(50), 4CzIPN(80) and 4CzIPN(100). Reaction conditions: 10 mg catalyst, 50 mL of 10% TA aqueous solution, 3 wt.% Pt, λ ≥ 420 nm, 10 °C.


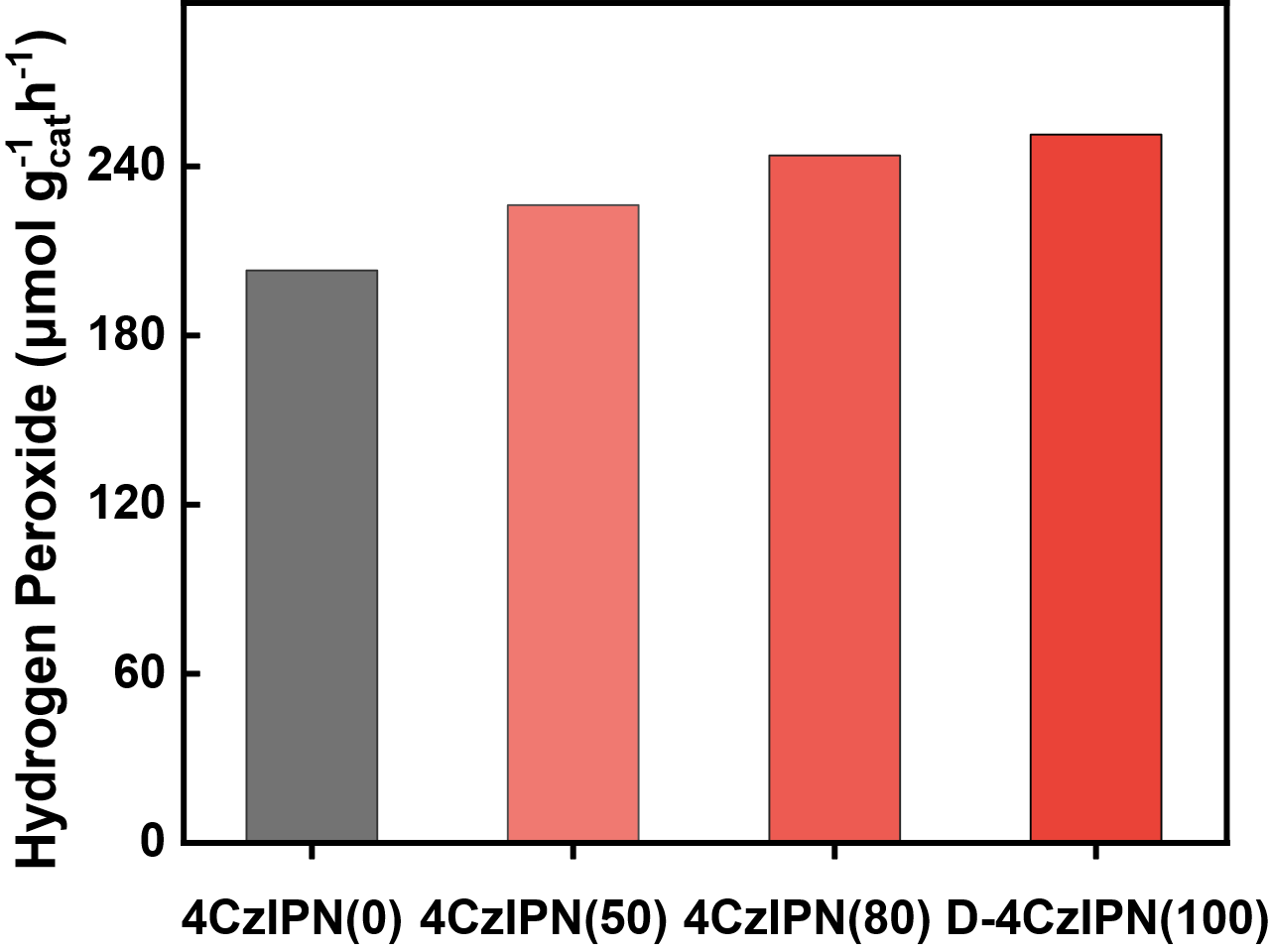
**Figure S25.** Photocatalytic hydrogen peroxide production tests of 4CzIPN(0), 4CzIPN(50), 4CzIPN(80) and 4CzIPN(100). Reaction conditions: 10 mg catalyst, 20 mL of 10% isopropyl alcohol (IPA) aqueous solution, O₂-saturated, λ ≥ 420 nm, 10 °C.

To verify the generality of the deuteration strategy, we additionally synthesized several organic photocatalysts, including 5CzBN, 6CzPh and their deuterated forms (D-5CzBN and D-6CzPh). Compared to 4CzIPN, these photocatalysts either bear donor-acceptor structures with different positions and numbers, or only bear donor units, providing us with a relatively diversified platform for investigating the generality of the deuterium strategy. These newly prepared organic photocatalysts were subsequently evaluated for photocatalytic hydrogen production (**Figure S26**) and photocatalytic hydrogen peroxide synthesis (**Figure S27**). It can be seen that the photocatalytic performance improved after deuteration for both 5CzBN and 6CzPh, indicating a strong generality of the deuteration strategy in enhancing the photocatalytic performance of carbazole-based organic photocatalysts.

**
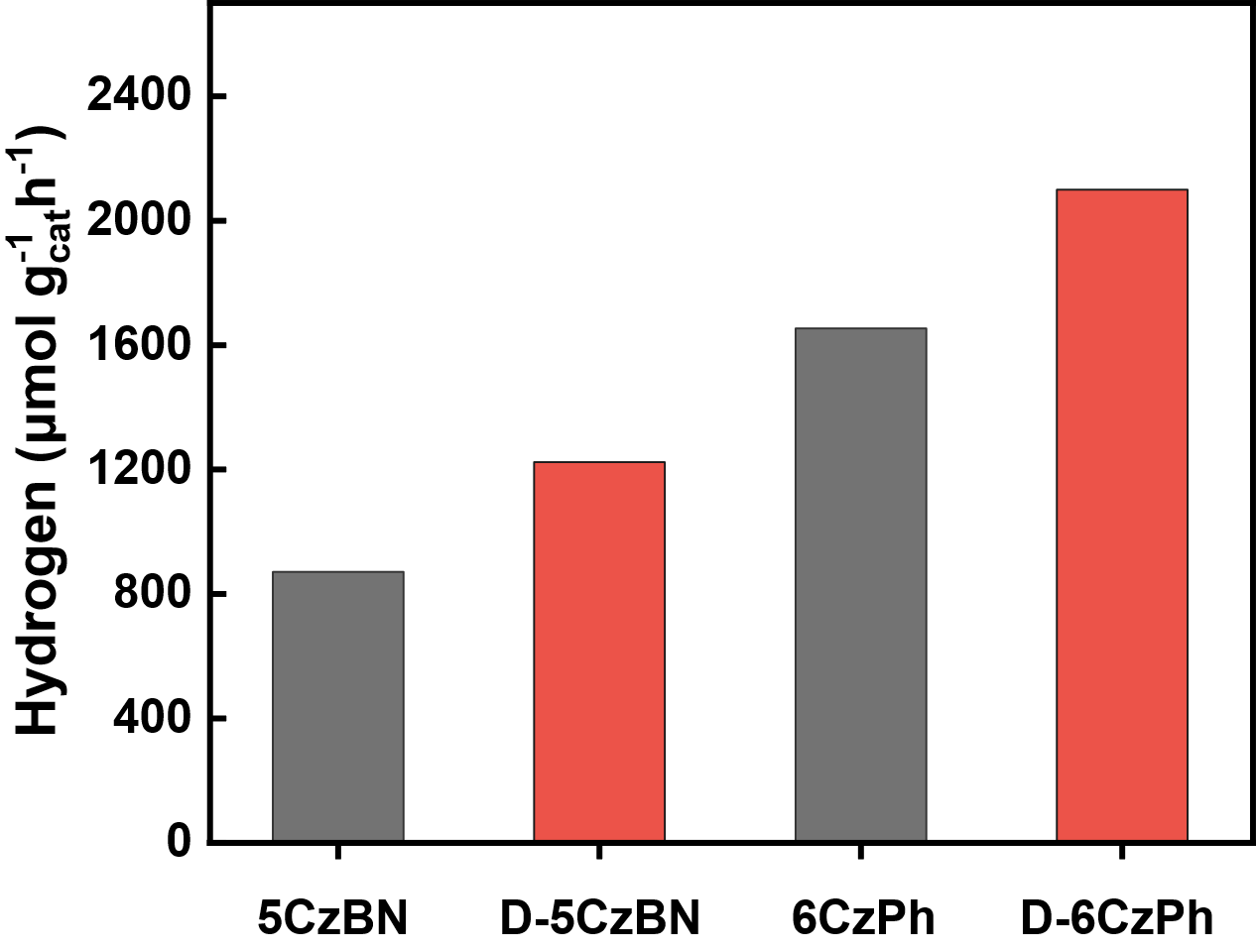
Figure S26.** Photocatalytic hydrogen evolution tests of 5CzBN, D-5CzBN, 6CzPh and D-6CzPh. Reaction conditions: 10 mg catalyst, 50 mL of 10% TA aqueous solution, 3 wt.% Pt, λ ≥ 420 nm, 10 °C.

**
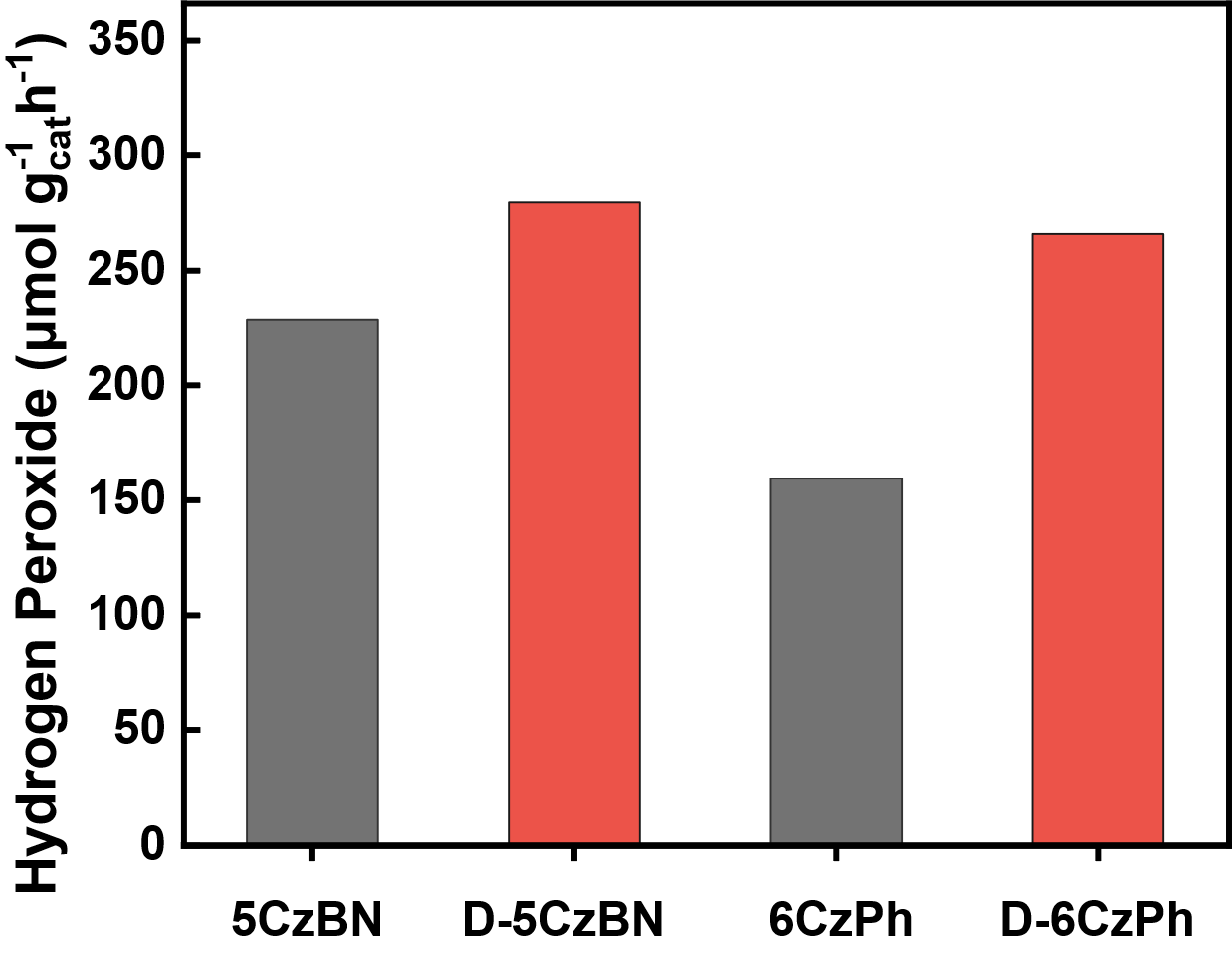
Figure S27.** Photocatalytic hydrogen peroxide production tests of 5CzBN, D-5CzBN, 6CzPh and D-6CzPh. Reaction conditions: 10 mg catalyst, 20 mL of 10% isopropyl alcohol (IPA) aqueous solution, O₂-saturated, λ ≥ 420 nm, 10 °C.

**
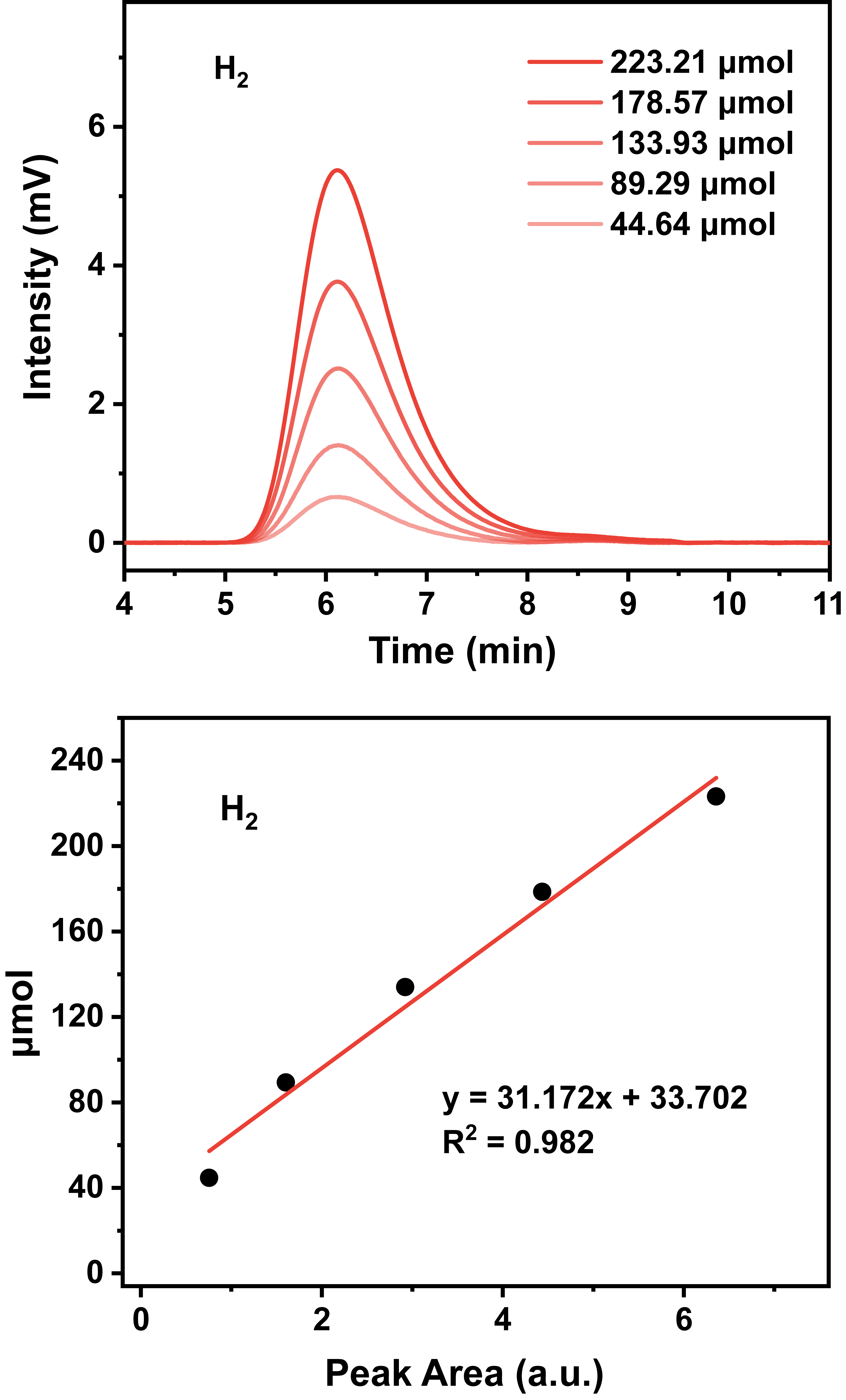
Figure S28.** H₂ calibration curve and its linear fit.

**Figure S29.** O₂ calibration curve and its linear fit**
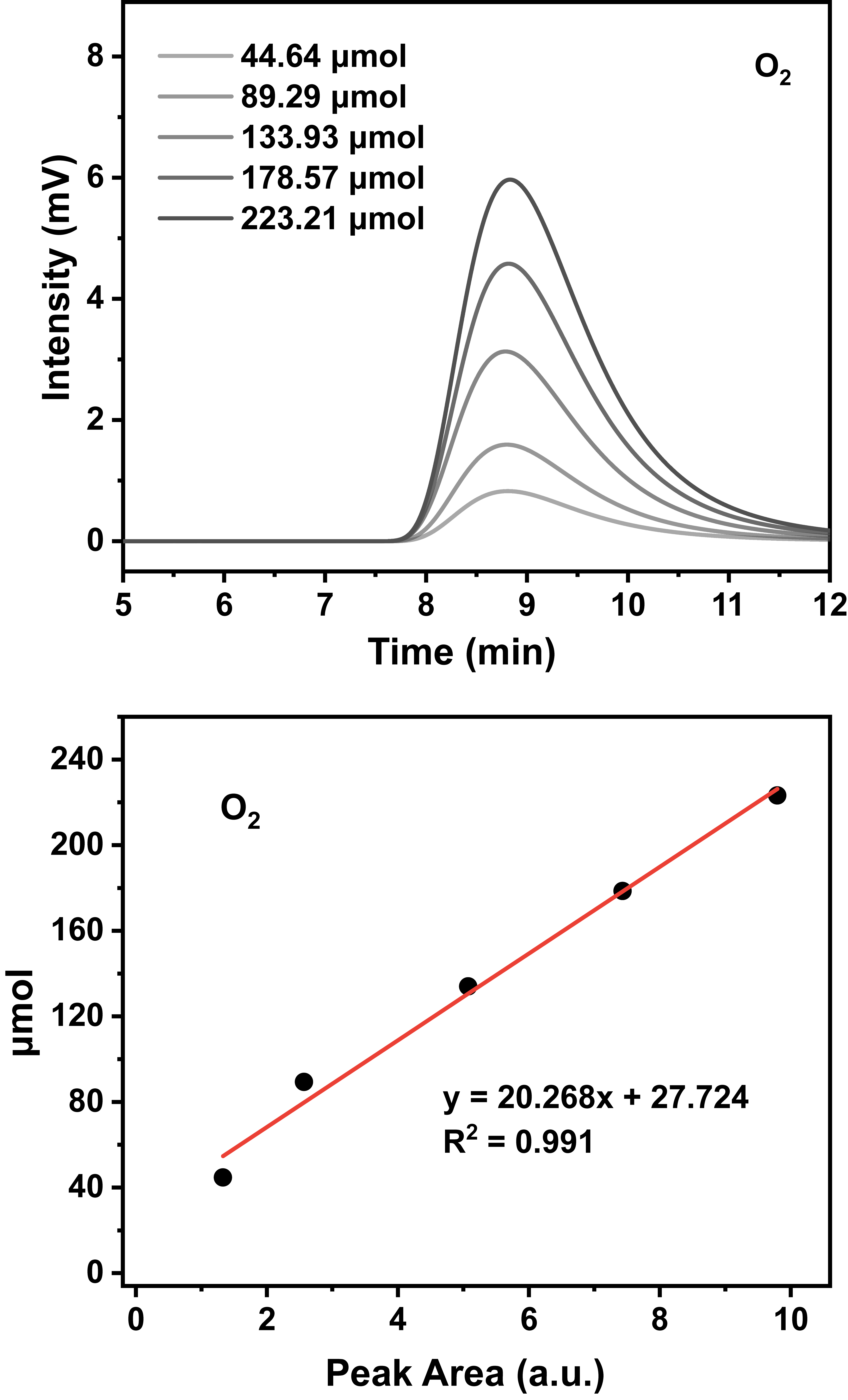
**.

1. The fitting parameters of TR-PL decay at 550 nm of 4CzIPN and D-4CzIPN in solid powder.

|  | **τ_1_(ns)** | **A_1_(%)** | **τ_2_(ns)** | **A_2_(%)** | **τ_aver_(ns)** |
| --- | --- | --- | --- | --- | --- |
| 4CzIPN | 13.81 | 80.85 | 203.25 | 19.15 | 50.08 |
| D-4CzIPN | 11.59 | 79.18 | 229.93 | 20.82 | 57.05 |

1. The fitting parameters of fs-TAs decay at 456 nm of 4CzIPN and D-4CzIPN in N_2_ degassed DMF solution.

|  | **τ_1_(ps)** | **A_1_(%)** | **τ_2_(ps)** | **A_2_(%)** | **τ_3_(ps)** | **A_3_(%)** | **τ_aver_(ps)** |
| --- | --- | --- | --- | --- | --- | --- | --- |
| 4CzIPN | 2.63 | 0.62 | 288.82 | 11.09 | 2687.28 | 88.29 | 2405.68 |
| D-4CzIPN | 0.72 | 0.95 | 12.08 | 4.55 | 245.71 | 94.5 | 232.76 |

1. The fitting parameters of TR-PL decay at 550 nm of poly-4CzIPN and D-poly-4CzIPN in solid powder.

|  | **τ_1_(ns)** | **A_1_(%)** | **τ_2_(ns)** | **A_2_(%)** | **τ_aver_(ns)** |
| --- | --- | --- | --- | --- | --- |
| 4CzIPN | 1.16 | 3.22 | 18.32 | 96.78 | 17.76 |
| D-4CzIPN | 1.82 | 4.87 | 19.73 | 95.13 | 18.86 |

1. The fitting parameters of fs-TAs decay at 485 nm of poly-4CzIPN and D-poly-4CzIPN in N_2_ degassed DMF solution.

|  | **τ_1_(ps)** | **A_1_(%)** | **τ_2_(ps)** | **A_2_(%)** | **τ_3_(ps)** | **A_3_(%)** | **τ_aver_(ps)** |
| --- | --- | --- | --- | --- | --- | --- | --- |
| 4CzIPN | 19.1 | 1.85 | 217.1 | 17.33 | 2148.6 | 80.82 | 1774.5 |
| D-4CzIPN | 14.8 | 0.28 | 244.1 | 23.91 | 751.0 | 75.81 | 627.7 |

The average lifetime (τ_aver_) of the active electron was determined using the equation:

$$\tau_{\mathrm{aver}}=\frac{\sum A_{i}\tau_{i}^{2}}{\sum A_{i}\tau_{i}}=\frac{(A_{1}\tau_{1}^{2}+A_{2}\tau_{2}^{2}+A_{3}\tau_{3}^{2}+\ldots)}{(A_{1}\tau_{1}+A_{2}\tau_{2}+A_{3}\tau_{3}+\ldots)}$$

**where** A_i_ **and** τ_i_ **refer to the pre-exponential factors and the corresponding electron lifetimes, respectively.**

**Supplementary Appendix**


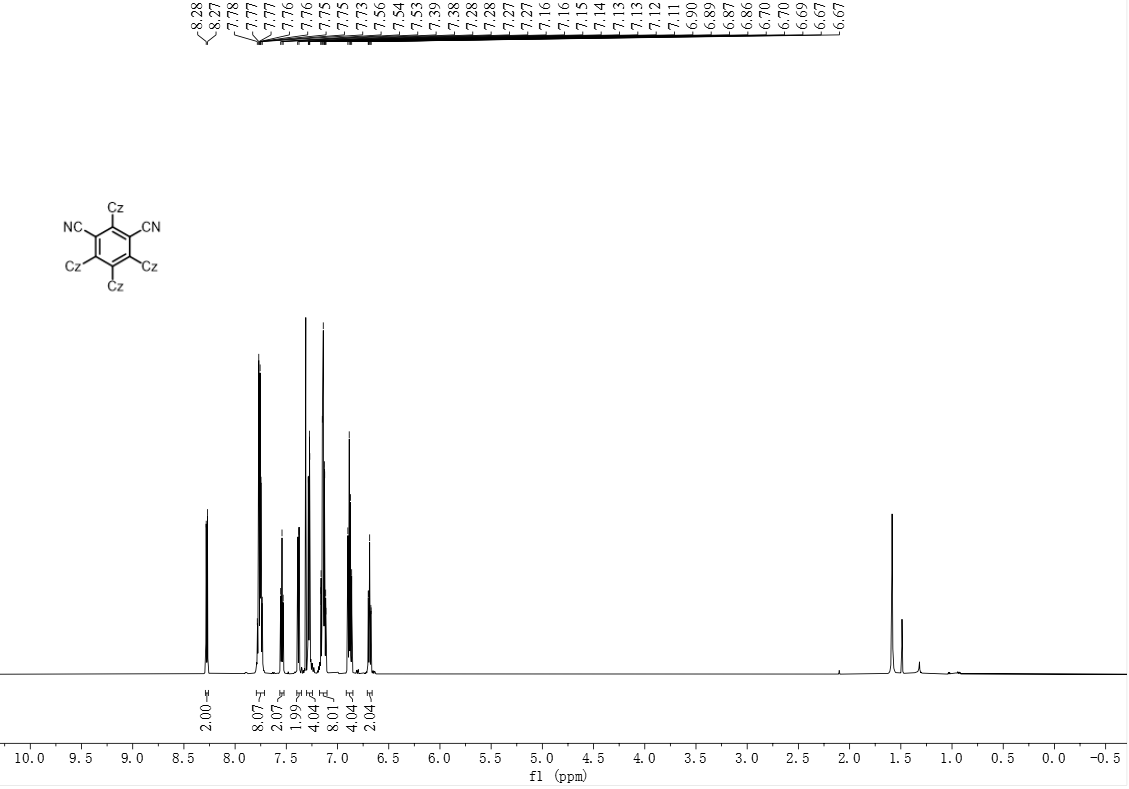


1. ^1^H-NMR of 4CzIPN monomer with CDCl_3_ as an internal standard.

4CzIPN monomer: ^1^H NMR (600 MHz, CDCl_3_) *δ* 8.29-8.26 (m, 2H), 7.75-7.65 (m, 8H), 7.56-7.52 (m, 2H), 7.40-7.35 (m, 2H), 7.30-7.25 (m, 4H), 7.18-7.10 (m, 8H), 6.92-6.85 (m, 4H), 6.71-6.66 (m, 2H) ppm.


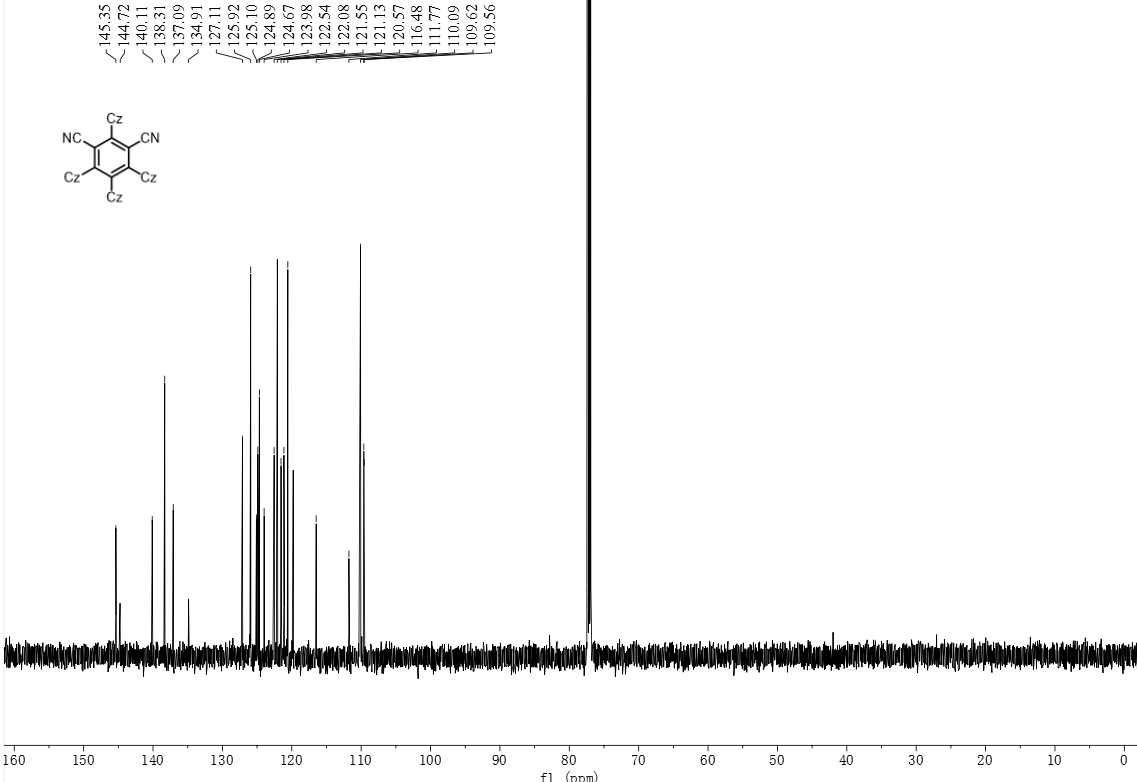


1. ^13^C-NMR of 4CzIPN monomer with CDCl_3_ as an internal standard.

4CzIPN monomer: ^13^C NMR (151 MHz, CDCl_3_) *δ* 145.4, 144.7, 140.1, 138.3, 137.1, 134.9, 127.1, 125.9, 125.1, 124.9, 124.7, 124.0, 122.5, 122.1, 121.6, 121.1, 120.6, 116.5, 111.7, 110.1, 109.62, 109.56 ppm.


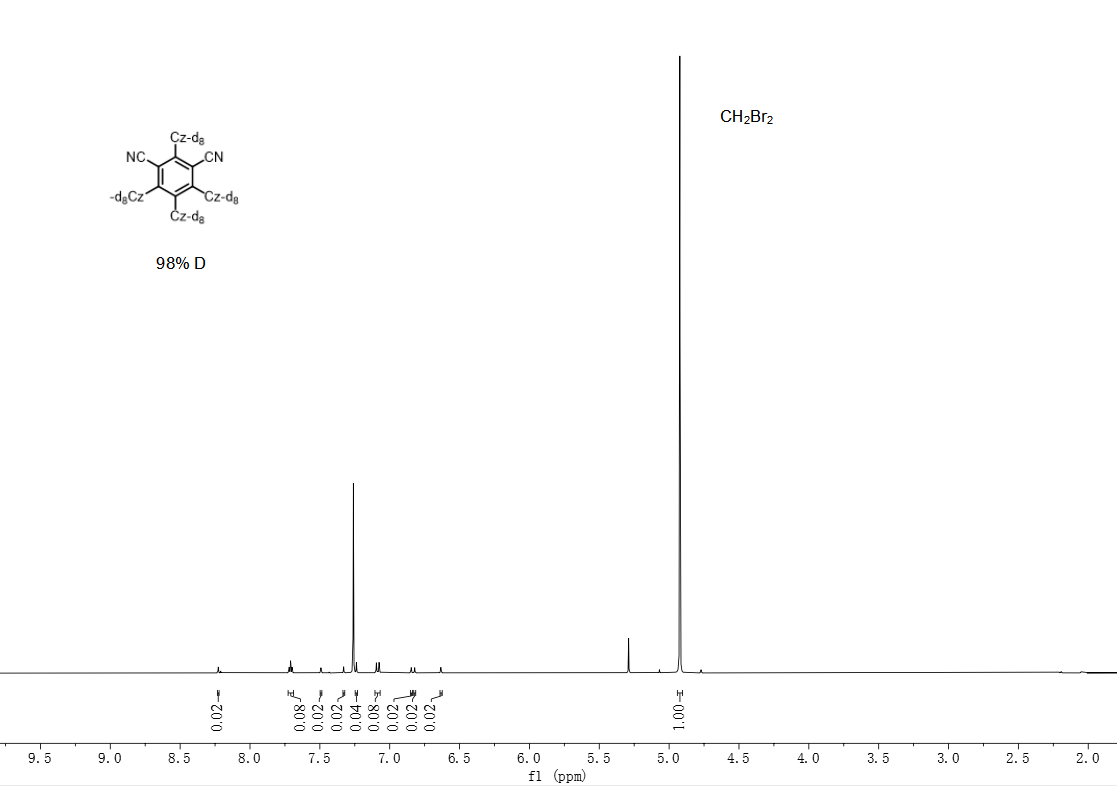


1. ^1^H-NMR of D-4CzIPN monomer with CDCl_3_ and CH_2_Br_2_ as an internal standard.

D-4CzIPN monomer：^1^H NMR (600 MHz, CDCl_3_) *δ* 8.27-8.26 (m, 0.02H), 7.77-7.73 (m, 0.08H), 7.54-7.53 (m, 0.02H), 7.38-7.36 (m, 0.02H), 7.29-7.27 (m, 0.04H), 7.15-7.11 (m, 0.08H), 6.89-6.86 (m, 0.04H), 6.68-6.66 (m, 0.02H), 4.96 (s, CH_2_Br_2_ 1.00H) ppm; The deuteration degree of deuterated 4CzIPN was determined to be 98% by adding the same molar amount bromomethane as an internal standard.


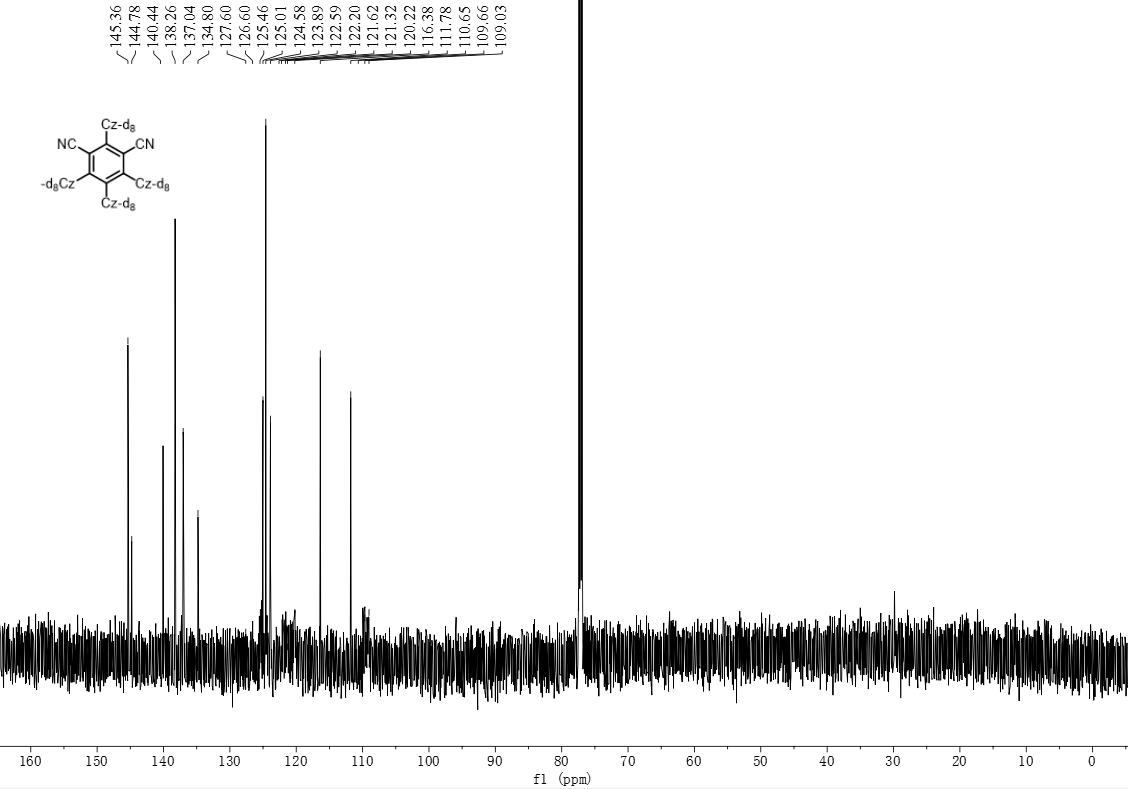


1. ^13^C-NMR of D-4CzIPN monomer with CDCl_3_ as an internal standard.

D-4CzIPN monomer：^13^C NMR (151 MHz, CDCl_3_) *δ* 145.4, 144.8, 140.4, 138.3, 137.0, 134.8, 127.6, 126.6, 125.5, 125.0, 124.6, 123.9, 122.6, 122.2, 121.6, 121.3, 120.2, 116.4, 111.8, 110.7, 109.76, 109.0 ppm.


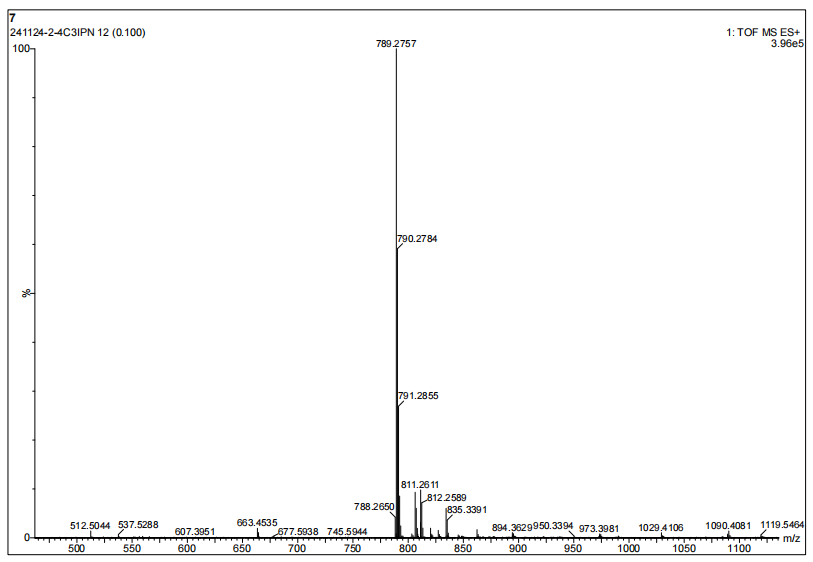


1. Mass spectrum of 4CzIPN monomer.

HRMS (ESI-TOF) m/z: [M+H]+ Calcd for C56H33N6Na+：789.2761; Found: 789.2757.


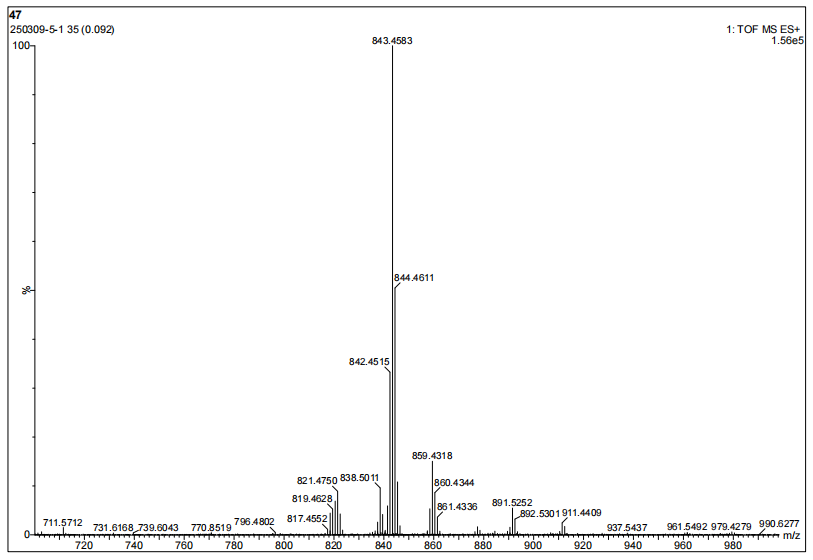


1. Mass spectrum of D-4CzIPN monomer.

HRMS (ESI-TOF) m/z: [M+H]+ Calcd for C56H33N6Na+：843.4589; Found: 843.4583。


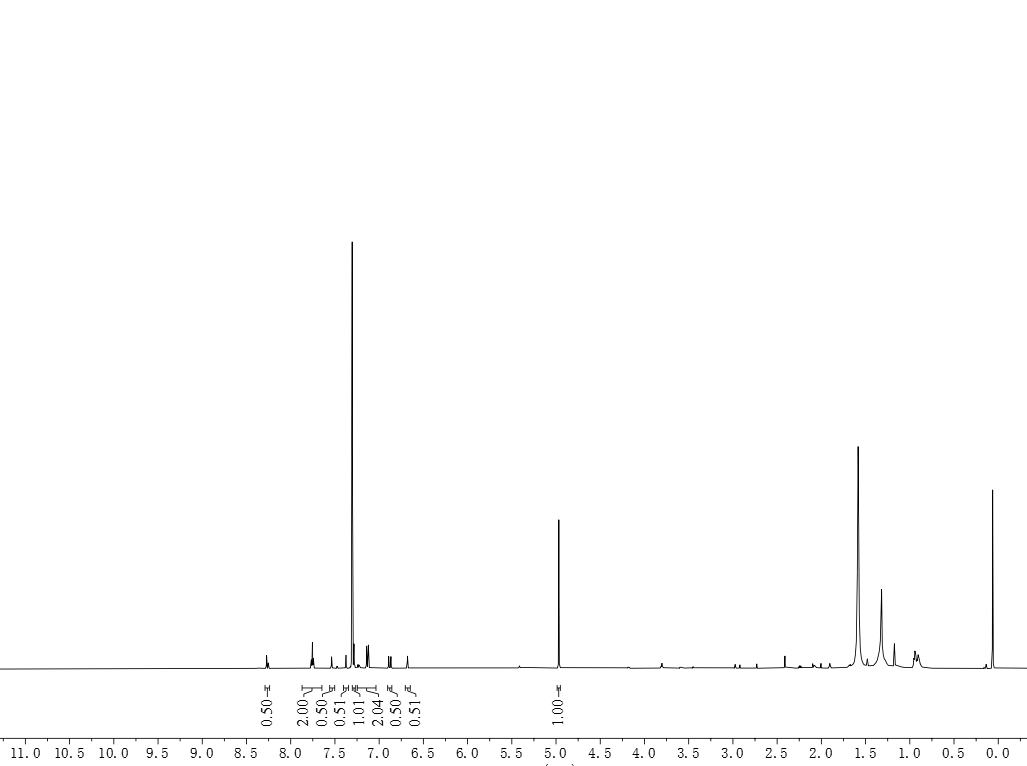


**Appendix S7** ^1^H-NMR of recovered D-4CzIPN monomer with CDCl_3_ and CH_2_Br_2_ as an internal standard. The deuteration degree of recovered D-4CzIPN was determined to be 98% using 1/25 equivalents of bromomethane as an internal standard.
